# Multi‐Frame Image Registration for Automated Ventricular Function Assessment in Single Breath‐Hold Cine MRI Using Limited Labels

**DOI:** 10.1002/mrm.70137

**Published:** 2025-10-18

**Authors:** Aya Ghoul, Paxstan Cassal Paulson, Andreas Lingg, Patrick Krumm, Kerstin Hammernik, Daniel Rueckert, Sergios Gatidis, Thomas Küstner

**Affiliations:** ^1^ Medical Image and Data Analysis (MIDAS.lab), Department of Interventional and Diagnostic Radiology University Hospital of Tuebingen Tuebingen Germany; ^2^ Institute of Signal Processing and System Theory University of Stuttgart Stuttgart Germany; ^3^ School of Computation, Information and Technology Technical University of Munich (TUM) Munich Germany; ^4^ TUM University Hospital Technical University of Munich (TUM) Munich Germany; ^5^ Department of Computing Imperial College London London UK; ^6^ Department of Radiology Stanford University Stanford California USA

**Keywords:** cine MRI, deep learning, image registration, motion‐compensated reconstruction, segmentation, strain

## Abstract

**Purpose:**

This study aims to develop an automated framework for operator‐independent assessment of cardiac ventricular function from highly accelerated images.

**Methods:**

We introduce a deep learning framework that generates reliable ventricular volumetric parameters and strain measures from fully sampled and retrospectively accelerated MR images. This method integrates image registration, motion‐compensated reconstruction, and segmentation in a synergetic loop for mutual refinement. The evaluation was performed on an in‐house dataset of healthy and cardiovascular‐diseased subjects. We examined the performance of the underlying tasks, including registration and segmentation, and their impact on derived parameters related to ventricular function.

**Results:**

The proposed approach demonstrates robustness to undersampling artifacts and requires limited annotation, while still reducing variability and errors for segmentation and registration. This translates to a 9% to 22% increase in Dice similarity compared to existing deep learning methods for left endocardium, left epicardium, and right ventricular delineation. Analysis of the predicted left and right ventricular ejection fraction reveals a strong correlation (>0.9) with manual measurements. Moreover, the estimated motion and segmentation masks enable consistent radial and circumferential strain measurements across accelerations up to R=24.

**Conclusion:**

A comprehensive ventricular function analysis can be performed using highly accelerated cine MR data with minimal annotation effort. This multitasking strategy has the potential to enable more accessible cardiac function analysis within a single breath‐hold.

## 1 Introduction

Cardiac cine Magnetic Resonance Imaging (MRI) captures the heart structure with high spatial and temporal resolution, enabling the quantification of regional function and the assessment of myocardial anatomy [1]. While volumetric parameters, such as ejection fraction, remain standard ventricular function indicators, they are inherently insensitive to regional dysfunction [2]. Conversely, myocardial strain provides global and regional insights into myocardial contractility and, therefore, a more comprehensive assessment of cardiac abnormalities [3, 4].

Deriving functional parameters, including volumetric measures and myocardial strain, from cine MRI data involves data acquisition over multiple breath‐holds, followed by image reconstruction, myocardial motion tracking, and image segmentation. The listed subtasks have recently become prominent areas of deep learning research, focusing on developing more generalizable and efficient methods [5, 6, 7, 8, 9, 10, 11, 12, 13]. The successful extraction of volumetric and strain measures relies heavily on the performance of the involved subtasks. The applied methods must be robust against low contrast, unclear myocardial borders, and variable intensities [14, 15].

Precise motion estimation and correction throughout the cardiac cycle are essential for a comprehensive assessment of ventricular function. Commonly, this is performed using pairwise image registration strategies, that is, registering frames to a reference or template frame [16, 17, 18], or aligning consecutive frames sequentially [19]. However, pairwise registration can be inaccurate for large cardiac motion [20] and sequential alignment can accumulate errors, causing a drift effect [21]. Additionally, these registration techniques frequently overlook the temporal context of neighboring frames. While previous approaches have enforced temporal smoothness using additional loss terms [22, 23], explicitly modeling temporal dynamics could better capture temporal features [24]. Therefore, encoding the motion evolution across the cardiac cycle can yield more temporally consistent and accurate motion estimations [25]. Moreover, accelerated acquisitions, used to reduce scan time, further complicate this task due to undersampling artifacts that obscure anatomical boundaries [19]. Motion priors have been integrated into reconstruction to jointly enhance motion estimation and image quality [23, 26], but their success depends on accurate registration, underscoring the need for robust motion estimation from accelerated data.

Ventricular segmentation is also essential to extract functional parameters. Recent deep learning segmentation networks have achieved state‐of‐the‐art results [12, 13, 27], but they often rely on large expert‐annotated datasets, restricting their applicability in studies with smaller cohorts [14, 28]. Publicly available datasets aim to address this challenge [28, 29]. However, cardiac data variability hinders creating universal methods, increasing the need for techniques that learn from limited annotations.

Ventricular analysis subtasks are often treated independently [30, 31] despite their inter‐dependencies. Enforcing consistency between segmentation and registration can boost both [32, 33]. While segmentation facilitates precise alignment of anatomical contours [11], registration propagates available manual masks to generate pseudo‐labels for unlabeled data [33]. Furthermore, reconstruction [6, 7, 8, 9] can provide high‐quality images from accelerated acquisitions to ensure reliable image registration and segmentation. In turn, motion information from image registration helps to solve the ill‐posed reconstruction problem by leveraging spatiotemporal context [26, 34, 35]. Combining image registration, segmentation, and reconstruction in a unified framework can improve the efficiency and accuracy of each subtask and thereby clinical parameter reliability [36].

In this work, we propose a deep learning‐based technique for automated left ventricular function characterization from fully sampled and accelerated cine MRI data. This approach is driven by the Motion Propagation Network (MOPNet), a novel multi‐frame image registration strategy. MOPNet addresses two key challenges: the scarcity of annotated data for training segmentation models and the requirement for accurate motion estimation from accelerated data to enable high‐quality motion‐compensated reconstruction under challenging conditions. The contributions of this study are threefold: (1) Our multi‐frame image registration approach extracts temporal dependencies across consecutive cardiac frames. Unlike existing groupwise methods that enforce temporal smoothness implicitly, we explicitly embed contextual temporal information to promote more consistent estimations. (2) We integrate registration, reconstruction, and segmentation to establish a continuous cycle of mutual refinement. This design reduces reliance on large annotated datasets, mitigates inconsistencies from separately trained models, and improves robustness across varying accelerations. (3) Our multitask method automatically analyzes ventricular function, encompassing volumetric measurements and local and global strain analysis directly from accelerated data. Moreover, the modular structure enables independent use of tasks during inference for flexible application.

We evaluated the proposed method in 36 retrospectively accelerated cases and identified a significant decrease in variability and errors in segmentation compared to other deep learning methods (Dice similarity increase of 9% to 22%). Furthermore, the consistent segmentation and registration performance enables reliable volumetric and strain measures extraction, even with single breath‐hold accelerated data. This work could therefore streamline cardiac imaging workflows and enhance patient comfort.

## 2 Methods

### 2.1 Joint Segmentation, Registration, and Motion‐Compensated Reconstruction Framework

**FIGURE 1:**
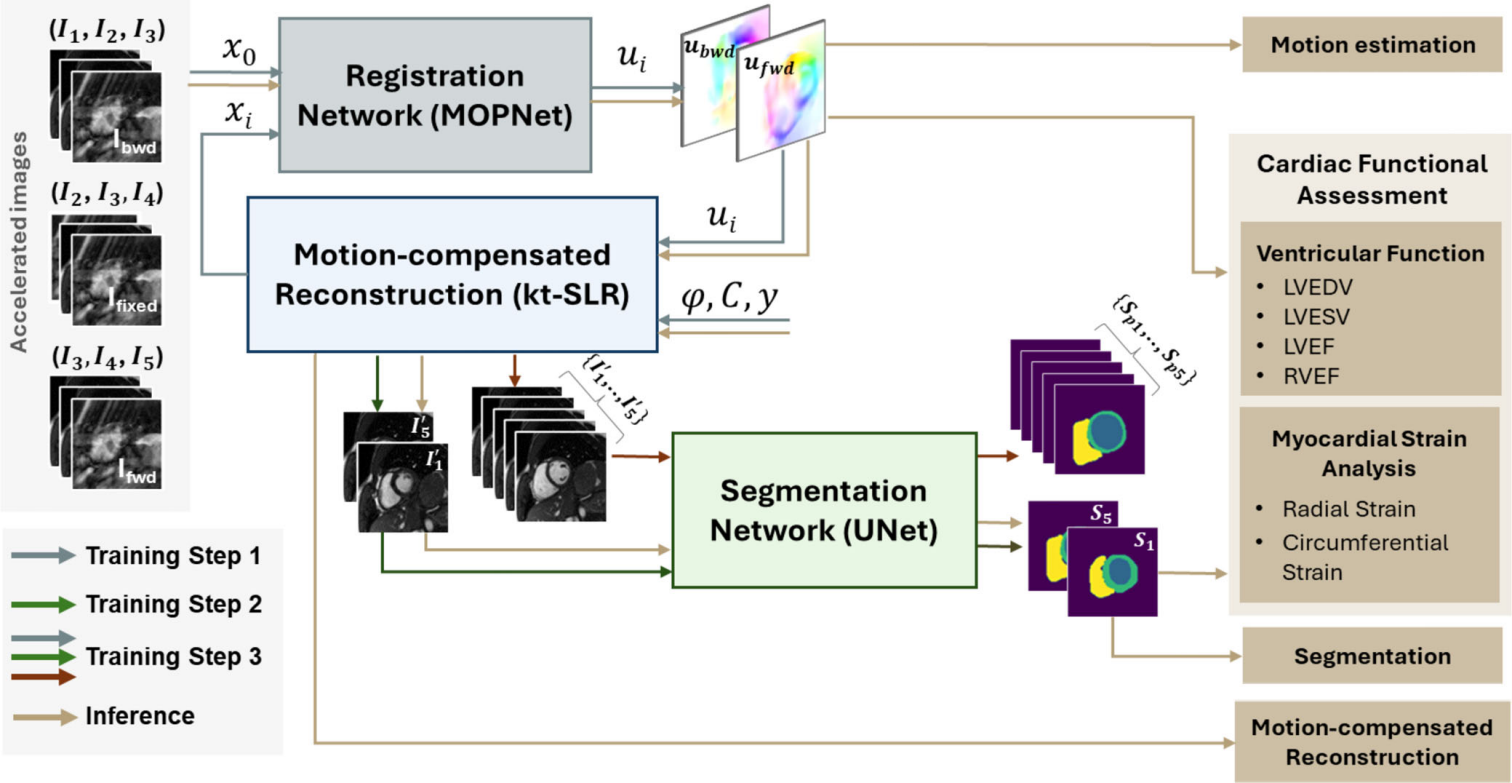
Proposed framework for joint registration, motion‐compensated reconstruction, and segmentation overview. A temporal sequence of five accelerated MR images, denoted as $I_{1}$, $I_{2}$, $I_{3}$, $I_{4}$, and $I_{5}$, is obtained retrospectively from the fully sampled k‐space y, the sensitivity maps C, and the undersampling mask ϕ. The registration and segmentation subnetworks are first trained independently. The reconstruction module is used to generate images $I_{1}^{\prime }$, $I_{2}^{\prime }$, $I_{3}^{\prime }$, $I_{4}^{\prime }$, and $I_{5}^{\prime }$ for training segmentation. Joint training then integrates reconstruction between registration and segmentation, with images iteratively refined via updated motion estimates $u_{i}$ to provide additional spatiotemporal context. Predicted segmentation masks S provide weak supervision for the registration network. Conversely, pseudo‐labels for unlabeled data are created based on motion estimates to train the segmentation network.

**FIGURE 2:**
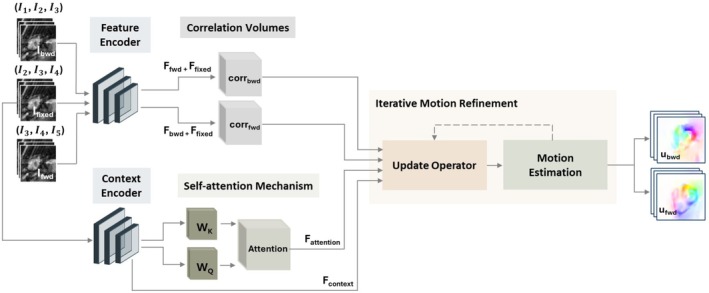
Proposed non‐rigid image registration network architecture. The input consists of tri‐frame groupings taken from five consecutive input images $I_{1}$ to $I_{5}$. Local spatial features $F_{bwd}$, $F_{\text{fixed}}$, and $F_{fwd}$ are extracted for the backward, fixed, and forward tri‐frame groups using a shared feature encoder. The context features $F_{\text{context}}$ are obtained via a dedicated context encoder. Feature similarities are stored in the forward $corr_{fwd}$ and backward $corr_{bwd}$ cost volumes and encoded to form the motion features. Self‐attention features $F_{attention}$ are derived by extracting query $W_{Q}$ and key $W_{K}$ maps from $F_{\text{context}}$. Residual motion updates are iteratively estimated by incorporating learned spatial and temporal representations using a GRU‐based update operator to output at the end the forward $u_{fwd}$ and backward $u_{bwd}$ motion estimates.

Efficient assessment of cardiac dynamics depends on robust motion tracking, handling of accelerated imaging, and precise segmentation. The proposed framework (Figure 1) integrates these interconnected steps through three key components: (i) a deep learning bidirectional image registration subnetwork, denoted MOPNet, to estimate motion from accelerated data (Figure 2); (ii) a motion‐compensated k‐t sparsity and low rank based reconstruction (kt‐SLR) [37] to leverage the spatiotemporal redundancies and thereby enhance the image quality, and (iii) a UNet [38] segmentation subnetwork to provide cardiac morphology annotation of the reconstructed images.

#### 2.1.1 Registration Subnetwork

The registration model is based on a tri‐frame optical flow (TROF) paradigm to capture motion dynamics across time. Unlike traditional pairwise [11, 19] or sequential registration methods [22, 23], we explicitly model bidirectional temporal dependencies to embed spatiotemporal representations [25]. The architecture comprises four stages: (1) tri‐frame grouping of the input images, (2) feature extraction and spatial correlation computation, (3) temporal motion propagation (MOP), and (4) motion estimation refinement.

##### 2.1.1.1 Tri‐frame Optical Flow

Let $I_{t}\in {\mathbb{R}}^{H\times W}$ denote the sequence of five consecutive temporal frames with spatial dimensions H×W, and t={1,2,3,4,5} indexes time. From this sequence, three overlapping tri‐frame groups are constructed [25]: the backward group $I_{\text{bwd}}=(I_{1},I_{2},I_{3})$, the fixed group $I_{\text{fixed}}=(I_{2},I_{3},I_{4})$ and the forward group $I_{\text{fwd}}=(I_{3},I_{4},I_{5})$. Our network matches the central tri‐frames $I_{\text{fixed}}$ to the preceding $I_{\text{bwd}}$ and succeeding frames $I_{\text{fwd}}$. The output consists of two motion estimates 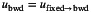 and 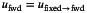, which represent dense pixel‐wise correspondences of shape (H,W,2) from each frame in the fixed frames $I_{\text{fixed}}$ to their three temporally adjacent frames in the backward and forward directions.

##### 2.1.1.2 Spatial Feature Extraction

The input triplets are reshaped to fuse the batch and temporal dimensions for efficient 2D convolution processing. Local spatial representations $F_{\text{bwd}}$, $F_{\text{fixed}}$ and $F_{\text{fwd}}$ are extracted individually using a shared feature encoder, while the context features $F_{\text{context}}$ are obtained via a dedicated context encoder [19]. A self‐attention mechanism builds the self‐similarities features of the fixed tri‐frames $F_{\text{attention}}$ based on the learned context representations [39]. Including local ($F_{\text{context}}$) and global ($F_{\text{attention}}$) features helps distinguish actual motion from aliasing in accelerated acquisitions during subsequent motion refinement stages [19].

Dual correlation volumes, denoted as $corr_{\text{bwd}}$ and $corr_{\text{fwd}}$, are precomputed using pairwise dot‐product similarities between the fixed features ($F_{\text{fixed}}$) and the backward ($F_{\text{bwd}}$) and forward ($F_{\text{fwd}}$) features [19, 40]. $corr_{\text{bwd}}$ encodes motion toward preceding frames, while $corr_{\text{fwd}}$ captures motion toward succeeding ones. These 4D volumes provide dense, spatial similarity maps for pixel‐level correspondence between the input images.

At each iteration k, we define a search neighborhood around each estimated pixel using multiscale correlation values $c_{\text{bwd,k}}$ and $c_{\text{fwd,k}}$, curated from the correlation volumes $corr_{\text{bwd}}$ and $corr_{\text{fwd}}$. We encode and stack the resulting correlation maps to create the correlation features $F_{{\text{corr}}_{k}}$. The current forward and backward estimates $u_{\text{bwd,k}}$ and $u_{\text{fwd,k}}$ are stacked to form the flow features, $F_{{\text{flow}}_{k}}$ [25, 40].

##### 2.1.1.3 Motion Propagation Module

The MOP module [25] enables temporal information sharing. Motion features are reshaped to restore the temporal dimension while preserving spatial and channel information. Each input triplet maintains a dedicated hidden motion state, denoted as $M_{\text{bwd}}$, $M_{\text{fixed}}$, and $M_{\text{fwd}}$, all initialized with a random state $M_{init}$. At each iteration k, neighboring motion states, $M_{k}^{-}$ and $M_{k}^{+}$, are retrieved from correlation features and warped to the spatial domain of the fixed frame using the current estimates $u_{\text{bwd},k}$ and $u_{\text{fwd},k}$ via a spatial transformer T:

(1) $$\begin{array}{l} M_{k}^{+}=T(M_{fwd,k}^{+};u_{fwd,k}) \end{array}$$

(2) $$\begin{array}{l} M_{k}^{-}=T(M_{bwd,k}^{-};u_{bwd,k}) \end{array}$$

$M_{k}^{+}$ and $M_{k}^{-}$ are subsequently concatenated with $M_{\text{fixed},k}$ to form the MOP feature:

(3) $$F_{\text{MOP}}^{k}=\text{Concat}(M_{fixed,k},M_{k}^{+},M_{k}^{-})$$

This representation is further encoded to update the hidden motion state and motion features:

(4) $$\begin{array}{lll} \left\{M_{fixed,k+1},F_{\text{motion},k}\}=\text{MotionEncoder}(F_{\text{corr,k}},F_{\text{flow,k}},F_{\text{MOP,k}}\right) & & \end{array}$$

Additionally, the motion states $M_{\text{fwd}}$ and $M_{\text{bwd}}$ are updated at each iteration by assigning the warped motion features:

(5) $$\begin{array}{l} M_{fwd,k+1}=M_{k}^{+} \end{array}$$

(6) $$\begin{array}{l} M_{bwd,k+1}=M_{k}^{-} \end{array}$$

Through recurrent updates, $M_{\text{fixed},k}$ progressively incorporates long‐range temporal information from neighboring image groups, effectively increasing its temporal receptive field across iterations [25].

##### 2.1.1.4 Update Module

The encoders that extract the correlation $F_{{\text{corr}}_{k}}$, flow $F_{{\text{flow}}_{k}}$ and MOP $F_{{\text{MOP}}_{k}}$ features are Superkernel blocks, used to ensure a large receptive field [41]. These building blocks use conical connections and depth‐wise convolutions to handle occlusions and capture representative spatial and temporal dependencies [25]. $F_{{\text{corr}}_{k}}$, $F_{{\text{flow}}_{k}}$ and $F_{{\text{MOP}}_{k}}$ are stacked and encoded into the motion features $F_{{\text{motion}}_{k}}$, then aggregated using $F_{\text{attention}}$ to create the motion aggregated features $F_{{\text{Agg}}_{k}}$ (Figure S1). $F_{{\text{motion}}_{k}}$, $F_{{\text{Agg}}_{k}}$ and $F_{\text{context}}$ are decoded with modified gated recurrent units (GRU) [19, 40] to output bidirectional residual flows $\Delta u_{k}$, consisting of $\Delta u_{\text{bwd,k}}$ and $\Delta u_{\text{fwd,k}}$. The residual flows are iteratively accumulated to refine the motion estimation, starting with zero initialization at all pixels.

##### 2.1.1.5 Inference

MOPNet receives a sequence of five consecutive frames as input and generates three bidirectional motion flows, mapping each frame of the fixed group to the forward and backward groups. Given the joint forward and backward training, the forward motion predictions inherently incorporate information from the backward tri‐frame group, promoting improved temporal coherence. Therefore, we consider only the forward motion estimates during inference. A sliding window strategy sequentially traverses the cyclic frame sequence to estimate motion between adjacent and distant frames. The stride within each window is progressively increased to capture motion at multiple temporal offsets. Window shifts are selected so that each frame pair is processed only once, avoiding redundant computation. This process is repeated until a complete frame‐to‐frame mapping of the entire cardiac cycle is determined.

#### 2.1.2 Motion‐Compensated Reconstruction

Motion‐compensated reconstruction provides high‐quality images that are better suited for the subsequent tasks, unlike the zero‐filled aliased images. We recover non‐rigid motion‐compensated images based on a generalized matrix formulation [42]. The acquired k‐space is modeled as the superposition of motion‐deformed views of T frames. Let $\tilde{y}$ denote the motion‐corrupted k‐space measurements for a given image x:

(7) $$\tilde{y}=\overset{T}{\underset{t=1}{\sum }}AU_{t}x$$

where A=ϕFC denotes the forward MR operation that includes the undersampling matrix ϕ, the Fourier transform F, and coil‐sensitivity maps C. The operator $U_{t}$ compensates for inter‐frame motion by aligning all T=5 input frames to a selected reference frame using motion estimations by MOPNet. The motion‐compensated k‐space y is then reconstructed by applying the kt‐SLR method [37] to the resulting sequence of T=5 aligned images.

#### 2.1.3 The Segmentation Network

The reconstructed images are segmented with a 2D U‐Net [38]. The contracting path has eight levels of 3×3 convolutions, with filter counts increasing from 32 to 512. Each layer is followed by ReLU activation and batch normalization. Max pooling is used for downsampling. In the expansive path, transposed convolutions upsample feature maps, which are then concatenated with corresponding features from the contracting path via residual connections. A 1×1 convolution with four filters generates a probabilistic segmentation mask, distinguishing the myocardium, right and left ventricular blood pools, and the background.

#### 2.1.4 Functional Assessment

MOPNet generates motion estimates for the entire cardiac cycle, which are used to perform the motion‐compensated reconstruction, including all T=25 frames. These reconstructed images are then processed again by MOPNet for motion refinement (i=1). While iterative refinement is possible, a single iteration yields sufficiently reliable motion estimates for the studied cases. The reconstructed images also serve as input to the segmentation network to produce precise annotations for the accelerated data.

The collected high‐quality segmented cardiac images with corresponding motion information enable detailed functional assessments. First, volumetric measurements, including left ventricular ejection fraction (LVEF) and right ventricular ejection fraction (RVEF), are calculated from the segmentation masks. Second, we quantify the radial and circumferential myocardial strains [43] based on the Green–Lagrangian strain tensor [44], given as:

(8) $$E=\frac{1}{2}\left(\nabla u+\nabla u^{⊤}+\nabla u^{⊤}\nabla u\right)$$

where u is the motion estimation provided by MOPNet as a function of position at end diastole and time. We establish a local coordinate system based on the epicardial surface determined from the end‐diastolic segmentation prediction. For each slice, a center of mass is computed from the epicardial contour. Radial unit vectors (r) are defined from the center of mass to each contour point, while circumferential vectors (c) are tangential and orthogonal to the radial directions along the contour. Both strains ($E_{rr}$ and $E_{cc}$) are computed by projecting the Green‐Lagrange tensor onto the corresponding unit direction as follows:

(9) $$\begin{array}{l} E_{rr}=r^{T}Er \end{array}$$

(10) $$\begin{array}{l} E_{cc}=c^{T}Ec \end{array}$$

For visualization, end‐systolic strain values are mapped onto the AHA 17‐segment polar map. Bull's eye plots are generated to display the regional distribution of systolic strain [45]. Additionally, strain maps overlaid on end‐systolic images provide a detailed visualization of localized variations based on pixel‐level myocardial deformation.

### 2.2 Datasets

We used 40 short‐axis 2D cine scans, including 20 healthy subjects (31±7 years, 6 female) and 20 patients (44±17 years, 9 female), with various cardiovascular diseases. The data were acquired in‐house on a 1.5T MRI (MAGNETOM Aera, Siemens Healthineers, Erlangen, Germany) with ECG gating. We used a vendor‐provided balanced steady‐state free processing (bSSFP) cine sequence with GRAPPA 2 acceleration. Left ventricular coverage was achieved over six breath‐holds of 16 s each, with two slices acquired per breath‐hold. The other imaging parameters were: TE/TR = 1.06/2.12 ms, flip angle = 52°, bandwidth = 915 Hz/px, spatial resolution = 1.9 × 1.9 mm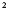 with a matrix size in the range of 176×132 to 192×180, slice thickness=8 mm, and 25 cardiac phases spanning the complete cardiac cycle, resulting in varying temporal resolutions of 34 to 58 ms across scans. Images were automatically delineated with the Segment software [46] and then manually corrected by an expert reader with over 10 years of cardiovascular MRI experience. The study was approved by the local ethics committee (426/2021BO1), and all the subjects gave written consent.

The dataset was partitioned into training and test subsets consisting of 36 subjects, along with a validation subset of 4 subjects. For all evaluations, fourfold cross‐validation was employed, where 75% of the subjects (27) were used for training and the remaining 25% (9) for testing in each fold. All images were zero‐padded to a grid of size 192×192 and normalized to values between −1 and 1 using min‐max normalization. During testing, center cropping was applied to recover the original shape.

The fully sampled k‐space data were retrospectively undersampled using the Variable Density Incoherent Spatiotemporal Acquisition (VISTA) technique [47]. Coil sensitivity maps were estimated from the acquired auto‐calibration signal data [48]. Zero‐filled coil‐weighted images were then obtained by applying an inverse Fourier transform to the masked k‐space data.

Inference was also performed on unseen data, including retrospectively accelerated radial scans [49], post‐contrast short‐axis scans from the in‐house dataset, and high‐resolution (1.5 × 1.5 mm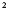) long‐ and short‐axis scans from the public CMRxRecon dataset [50].

### 2.3 Training and Implementation

The registration and segmentation networks were initially trained independently, with one network being frozen while the other was optimized (Figure S2). The registration network was trained with a self‐supervised strategy based on the photometric loss ${ℒ}_{photometric}$ to measure the similarity between the fixed images $I_{\text{fixed}}$ and the warped versions of the backward $I_{\text{bwd}}$ and forward $I_{\text{fwd}}$ images using the motion estimate:

(11) $$\begin{array}{ll} {ℒ}_{\text{photometric}} & ={\left\|I_{\text{fixed}}-T(I_{\text{bwd}},u_{\text{bwd}})\right\|}_{1}\\ & \;+{\left\|I_{\text{fixed}}-T(I_{\text{fwd}},u_{\text{fwd}})\right\|}_{1} \end{array}$$

Additionally, the smoothness loss ${ℒ}_{smoothness}$ encouraged locally smooth motion fields in $u_{bwd}$ and $u_{fwd}$ [19]:

(12) $${ℒ}_{\text{smoothness}}={\left\|\nabla u_{bwd}\right\|}_{1}+{\left\|\nabla u_{fwd}\right\|}_{1}$$

The registration training loss [19] (${ℒ}_{reg}$) optimized the predictions for K=6 iterations:

(13) $${ℒ}_{reg}=\overset{K=6}{\underset{k=1}{\sum }}{ℒ}_{photometric,k}+\lambda_{1}{ℒ}_{smoothness,k}$$

with $\lambda_{1}=0.04$. MOPNet was trained for 150 000 training steps. Then, the segmentation network was trained on fully sampled labeled data using a soft multi‐class Dice loss ${ℒ}_{Dice}$ [33] for 50 000 training steps.

Both networks were subsequently optimized jointly while incorporating the motion‐compensated reconstruction. Manually labeled end‐systolic and end‐diastolic frames served as either the first frame in $I_{\text{bwd}}$ or the last in $I_{\text{fwd}}$ to enable pseudo‐label generation via motion warping. Intermediate frames ($I_{2}$ to $I_{4}$) were randomly selected with increasing temporal order. During standalone MOPNet training and inference, all frames were sampled arbitrarily.

To exploit unlabeled data, manual end‐systolic and end‐diastolic segmentations were warped to intermediate frames using our motion estimates. These warped masks served as pseudo‐labels and were incorporated into the training process via an additional multi‐class Dice loss (${ℒ}_{Dice-warp}$) to enable learning from labeled and unlabeled data. The total joint training loss (${ℒ}_{joint}$) is given as

(14) $${ℒ}_{joint}={ℒ}_{reg}+\lambda_{2}{ℒ}_{Dice}+\lambda_{3}{ℒ}_{Dice-warp}$$

with $\lambda_{2}=0.05$ and $\lambda_{3}=0.04$. The joint model was trained for 200 000 training steps. All models were optimized using the AdamW optimizer [51] (batch size = 4, learning rate = $1\times 10^{-4}$, weight decay=$5\times 10^{-4}$) on one NVIDIA V100 GPU with conservative learning rates, a cosine annealing schedule [52] and careful regularization to ensure robustness against transient inaccuracies. Hyperparameters were tuned via grid search to maximize the Dice similarity coefficient (DSC) on the validation dataset.

### 2.4 Experiments

#### 2.4.1 Segmentation, Registration, and Reconstruction Performance

We evaluated the predicted segmentation masks at end‐systole and end‐diastole, using DSC, the Hausdorff distance (HDD), and the Mean Contour Distance (MCD) for the fully sampled case and different accelerations. Results were benchmarked against two state‐of‐the‐art segmentation networks, nnUNet [12] and FCT [13], and two joint registration and segmentation frameworks, MotionNet [32] and DeepAtlas [33]. We trained the baselines using fully sampled data and adopted the hyperparameter settings recommended in their respective publications. These parameters provided the best DSC on the validation dataset after grid search‐based hyperparameter tuning. Furthermore, we evaluated the accuracy of the volumetric measures derived from the predicted segmentations, including LVEF and RVEF. Finally, we compared the predicted contours overlaid on the fully sampled images against the corresponding manual segmentation qualitatively.

We assessed image registration performance at various accelerations using the overlap score (OS), defined as the DSC between manual and propagated masks using motion estimation. Manual left and right ventricle labels were warped between end‐diastole and end‐systole using the estimated motion. We also computed the normalized root mean square error (NRMSE) between the warped moving image and the fixed image.

The percentage of nonpositive Jacobian determinants, indicating folding or unrealistic deformations, was reported to assess motion smoothness and anatomical plausibility. A 0% rate is desired to ensure a diffeomorphic transformation and preserved topology. Finally, we qualitatively examined the impact of acceleration by visualizing color‐coded [53] motion estimates and quiver plots overlaid on the fully sampled moving image.

We evaluated the reconstruction using the structural similarity index measure (SSIM), NRMSE, and qualitative comparison with fully sampled references.

#### 2.4.2 Ablation Study

We performed multiple ablation studies to assess the impact of key modules on segmentation and registration using fully sampled and R=24 accelerated data. We reported the DSC for segmentation and OS for registration, calculated for the right and left ventricular blood pools. Model variants selectively removed the reconstruction (w/o recon), registration (w/o reg), and segmentation (w/o seg) modules, individually or in combination. We also compared MOPNet to pairwise alignment without reconstruction (w/o TROF + w/o recon) and motion‐compensated kt‐SLR to conventional kt‐SLR to assess the effect of motion integration on image quality.

#### 2.4.3 Statistical Analysis

We reported the mean and standard deviation across the slices of the entire cohort obtained based on fourfold cross‐validation. Statistical significance was assessed via one‐way analysis of variance (ANOVA), followed by Tukey's honestly significant difference (HSD) test. Bonferroni/Holm post‐hoc corrections were applied for all pairwise group comparisons. A p‐value ≤0.05 was considered statistically significant with adjustments for multiple comparisons. We used Bland‐Altman analysis to quantify the agreement between predicted and manual volumetric measures. Bias and standard deviation were computed from differences between predicted and manual volumes. Limits of agreement (LoA) are defined as the bias ±1.96× the standard deviation. Regression plots and Pearson's correlation coefficients (r) were used to assess agreement in performance between manual and predicted LVEF and RVEF and across accelerations for global strain measures.

## 3 Results

### 3.1 Fully Sampled Data

**FIGURE 3:**
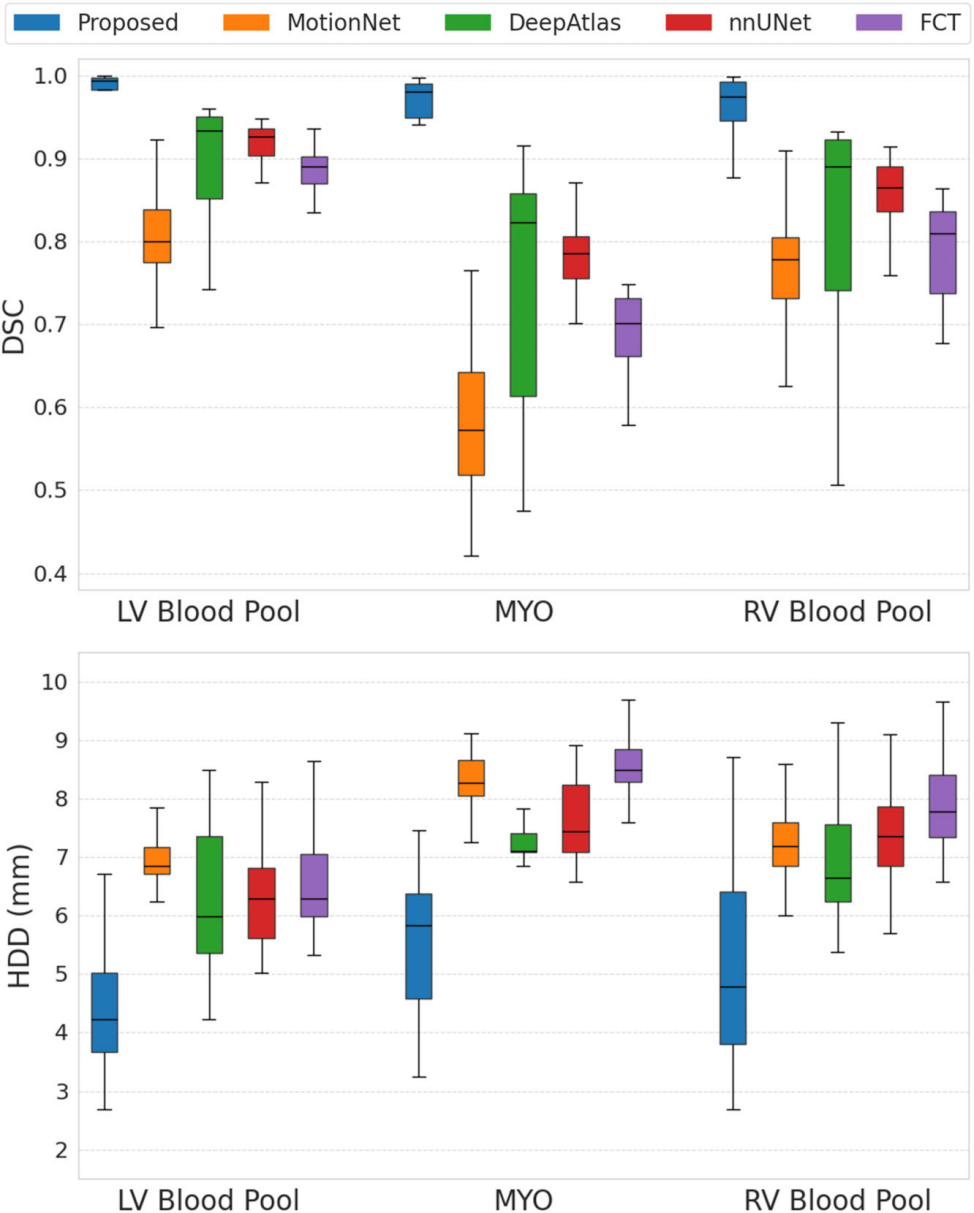
Boxplots (horizontal line: median, box: 25th/75th percentile, standard deviation: whiskers) of Dice scores (DSC), and Hausdorff Distances (HDD) obtained using our method compared to MotionNet [32], DeepAtlas [33], nnUNet [12], and FCT [13]. Metrics are obtained from fully sampled data using fourfold cross‐validation. We achieved improved performance compared to competing models for the right (RV) and left (LV) ventricular blood pools and the myocardium (MYO).

**FIGURE 4:**
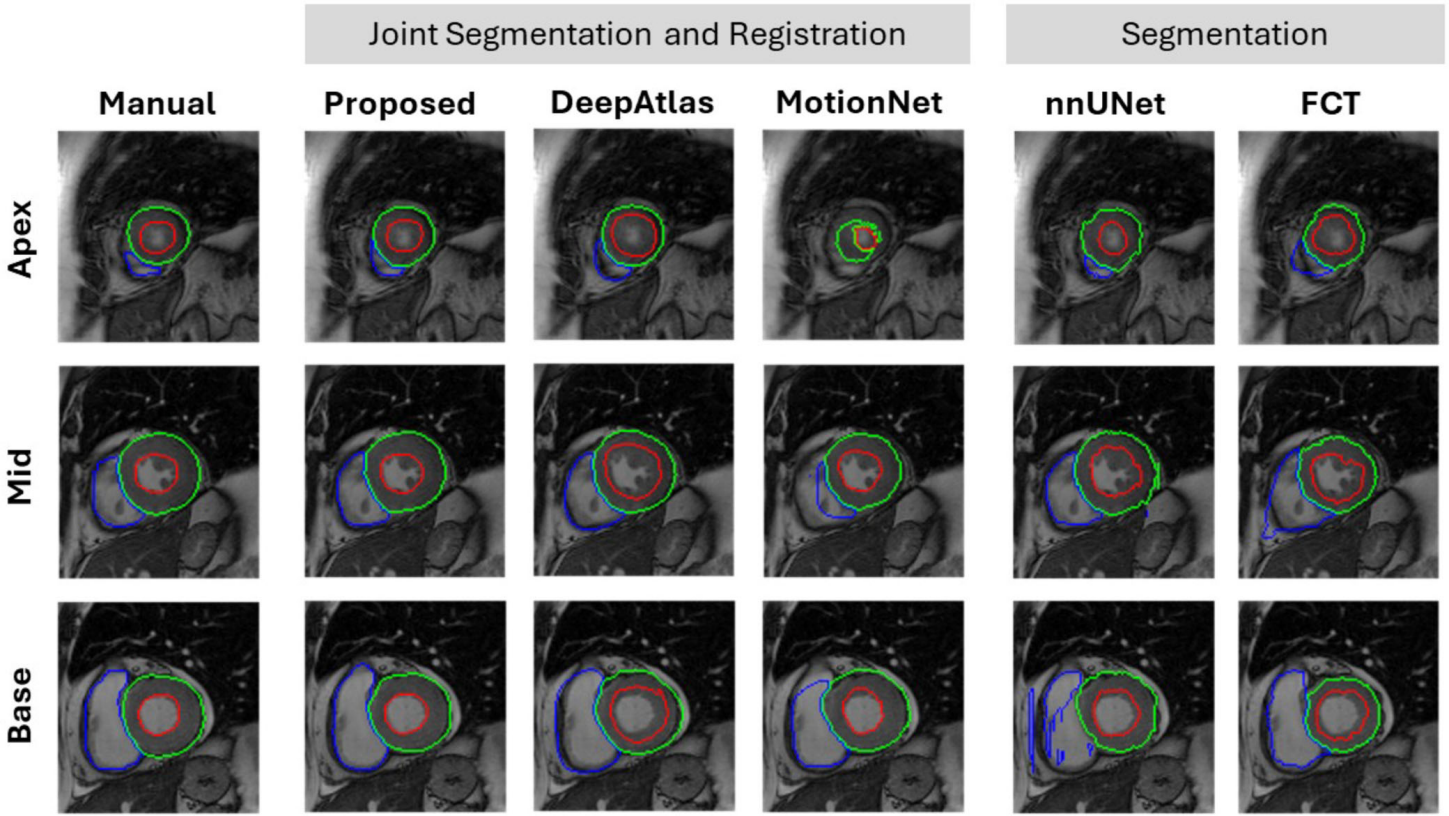
End‐systolic segmentation of the left and right ventricles, including apical, mid‐ventricular, and basal segments overlaid on short‐axis cine images. Results from the proposed method were compared to those of the segmentation networks (nnUNet [12], FCT [13]) and the joint segmentation and registration competing methods (MotionNet [32], DeepAtlas [33]). The other methods exhibited inaccurate delineations, observed as misaligned boundaries. In contrast, our approach achieved superior segmentation accuracy with well‐aligned contours.

The quantitative segmentation results demonstrated the superior performance of the proposed method compared to other deep learning segmentation models on fully sampled data (Figure 3). We achieved a higher average DSC score, with improvements between 9% to 22%, and a lower average HDD value, reduced by 1.7 to 2.6 mm. Qualitative assessment during systole and diastole further confirmed these findings. Figure 4 and Figure S3 show comparative segmentation results for apex, mid‐ventricular, and basal slices obtained in a healthy subject. The segmentation predictions provided by our strategy aligned closely with the manual annotations. In contrast, competing methods frequently exhibited inaccuracies, such as over‐segmentation or under‐segmentation of the right ventricle. We also observed poorly defined myocardial contours, especially in the apex and basal regions. The nnUNet model occasionally produced anatomically implausible or fragmented segmentation masks.

**FIGURE 5:**
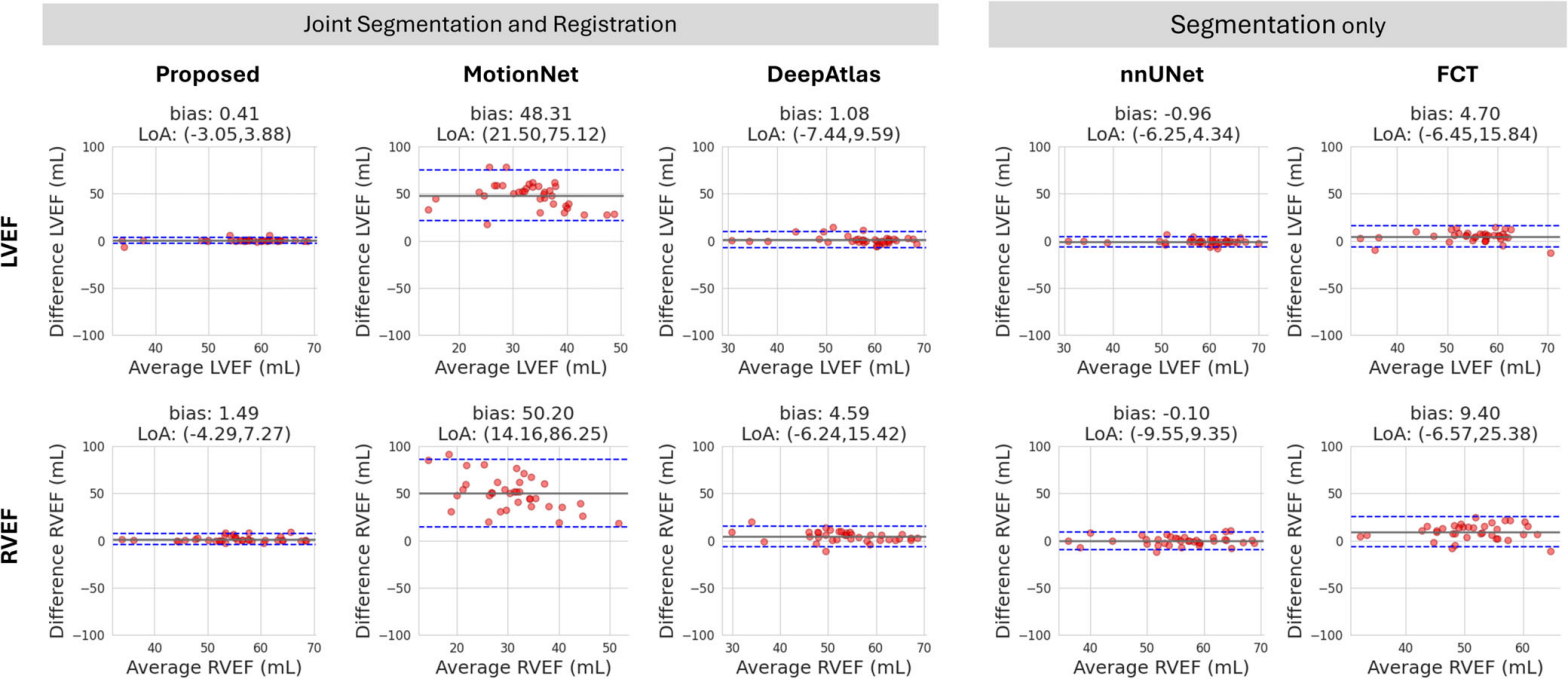
Bland‐Altman plots of manual versus automated assessments of the left ventricular (LVEF) and the right ventricular (RVEF) ejection fraction comparing the proposed method to segmentation networks (nnUNet [12], FCT [13]) and joint segmentation and registration networks (MotionNet [32], DeepAtlas [33]). The solid gray line indicates the mean difference (bias) and the dashed blue lines represent the limits of agreement (LoA, ±1.96 dollar standard deviations from the mean). Our method produced the highest correlation with the manual measures and the narrowest limits of agreement.

We examined the Bland‐Altman plots of the LVEF and RVEF to assess the clinical impact of our improved segmentation results (Figure 5). Our method showed the strongest correlation with manual measurements, with mostly smaller biases and tighter confidence intervals. Competing methods, especially MotionNet, showed wider limits of agreement, higher variability, and inaccuracies in both ejection fraction estimates.

### 3.2 Accelerated Data

**FIGURE 6:**
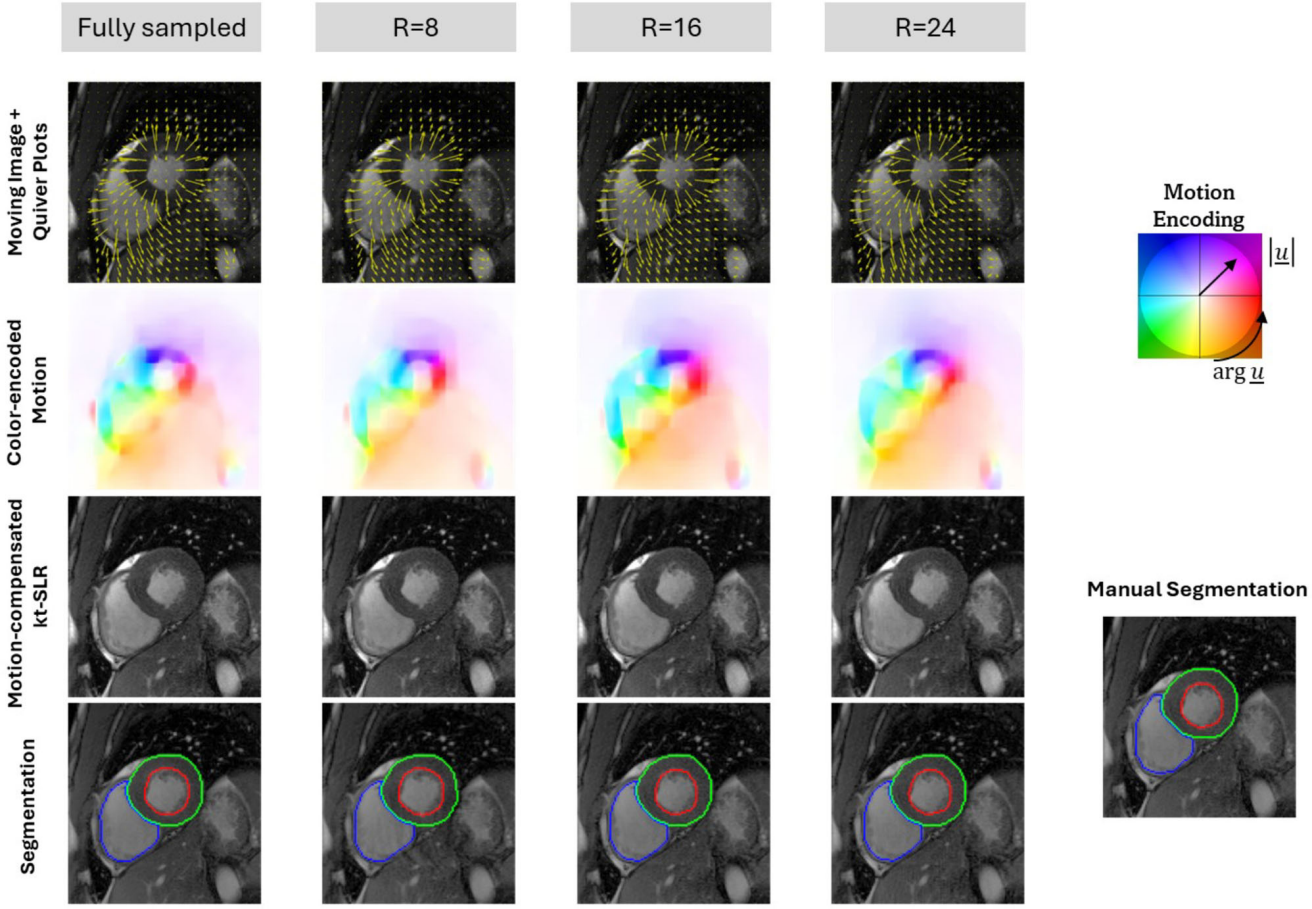
Representative motion estimates between end‐systolic (ES) and end‐diastolic (ED) cardiac cine frames, motion‐compensated reconstruction, and segmentation at end‐systole obtained with the proposed framework from a healthy subject. Accelerated data is obtained with retrospective undersampling using the VISTA mask at three accelerations (R=8, R=16, and R=24). Results are represented with quiver plots overlaid on the fully sampled moving image (first row), color‐encoded [53] motion estimates (second row), motion‐compensated reconstruction using kt‐SLR (third row), and segmentation contours (last row) overlaid on the corresponding fully sampled images.

The robustness of the motion estimation, motion‐compensated reconstruction, and segmentation towards different accelerations in a healthy subject is depicted in Figure 6 and Figure S4. The quiver plots and color‐encoded motion displacements showed that the underlying cardiac motion patterns are correctly represented during relaxation and contraction. The motion estimates remained consistent with a static background and exhibited no boundary ambiguities, even under high acceleration. The obtained motion‐compensated reconstructions of the end‐systolic and end‐diastolic frames closely aligned with the fully sampled references, featuring sharp edges and minimal residual artifacts. The segmentation masks indicated precise delineation of the cardiac structures across different accelerations.

**FIGURE 7:**
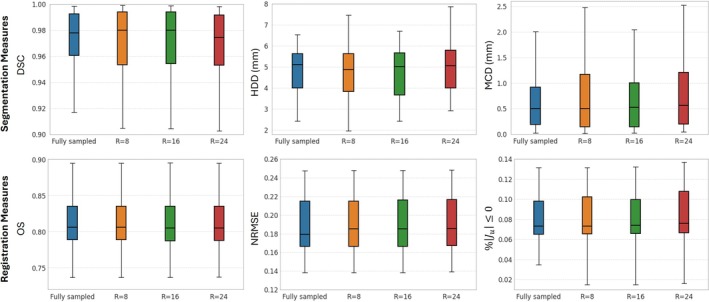
Boxplots (horizontal line: median, box: 25th/75th percentile, standard deviation: whiskers) of segmentation (top) and registration (bottom) for quantitative analysis of the proposed method on fully sampled, R=8, R=16, and R=24 accelerated data using the VISTA mask. For segmentation, the DSC, the Hausdorff Distance (HDD), and the Mean Contour Distance (MCD) are shown. The registration metrics include the OS, the normalized root‐mean‐square error (NRMSE), and the percentage of nonpositive Jacobian determinant values ($\%|J_{\phi }|\leq 0$). The metrics remain consistent across the different accelerations for both tasks, demonstrating the robustness of the proposed method in handling varying levels of acceleration.

Quantitative performance comparisons between the fully sampled case and accelerated cases (up to R=24) for cardiac motion estimation and segmentation performance remained stable across all accelerations (Figure 7). Statistical analysis confirmed no significant differences in DSC (F‐statistic = 0.059, p‐value = 0.981), HDD (F‐statistic = 0.188, p‐value = 0.904), and MCD (F‐statistic = 0.001, p‐value = 0.909). The high p‐values (all >0.05) and the low F‐statistics suggested that inter‐subject variability dominated over differences between acceleration rates. Similarly, statistical analysis revealed no significant differences in registration metrics across different acceleration rates. OS (F‐statistic = 0.001, p‐value = 0.998), NRMSE (F‐statistic = 0.027, p‐value = 0.993), and $\%|J_{\phi }|\leq 0$ (F‐statistic = 0.111, p‐value = 0.953) demonstrated negligible variability.

Quantitative evaluation results of the proposed reconstruction method are presented in Figure S5. The motion‐compensated kt‐SLR approach demonstrates consistently strong reconstruction performance across varying acceleration factors. SSIM remains high, with values of: SSIM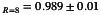, SSIM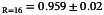, SSIM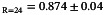. Corresponding NRMSE values are NRMSE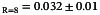, NRMSE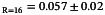, NRMSE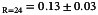. These results indicate that our method maintains high fidelity to the fully sampled reference images at higher accelerations. The qualitative results (Figures 6 and S4) show that the reconstruction closely resembles the fully sampled images, with minimal visible artifacts and well‐preserved anatomical structures.

**FIGURE 8:**
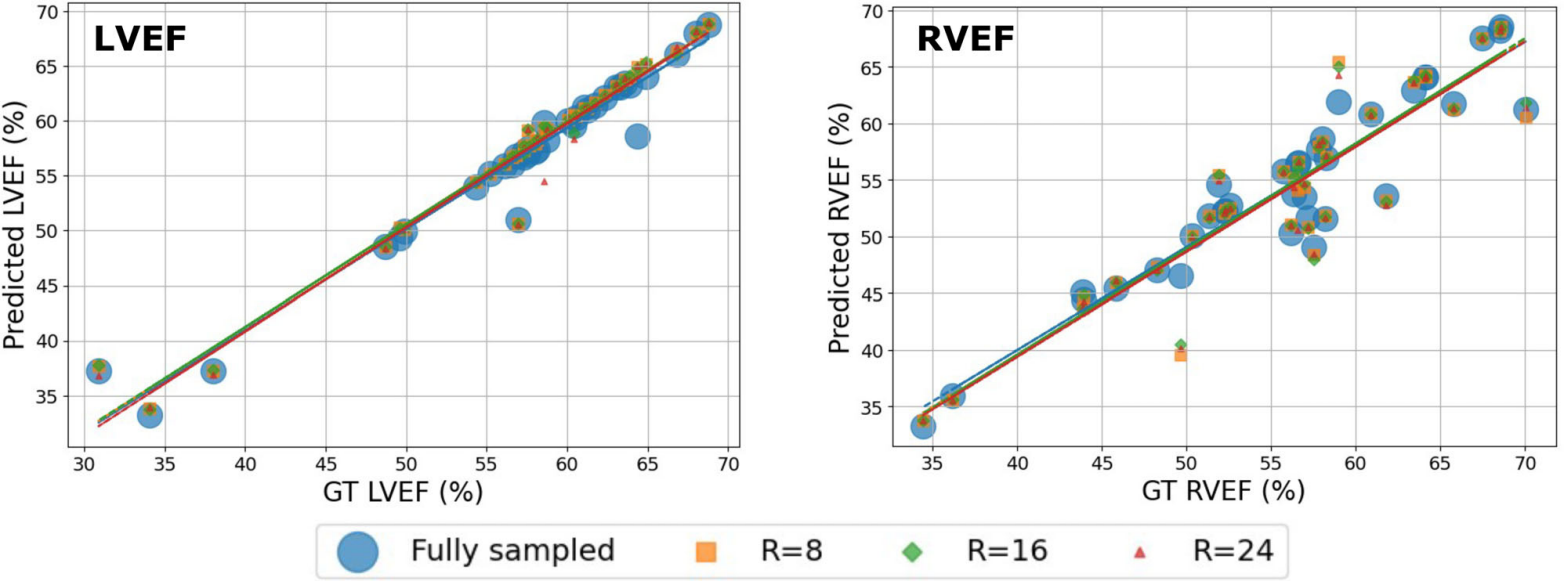
Linear regression plots illustrate performance differences between manually derived and predicted volumetric measures for Left Ventricular (LVEF) and Right Ventricular (RVEF) Ejection Fraction. Values are shown for the fully sampled case and the accelerations R=8, R=16, and R=24. Our method maintains high agreement with manual measures despite varying acceleration factors.

LVEF and RVEF derived from manual annotations were strongly correlated (Figure 8) across all subjects with those predicted at the fully sampled case and the accelerations R=8, R=16, and R=24 (LVEF: r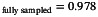, r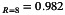, r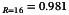, r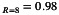, p‐value = 0.999, RVEF: r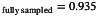, r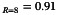, r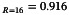, r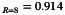, p‐value = 0.903).

**FIGURE 9:**
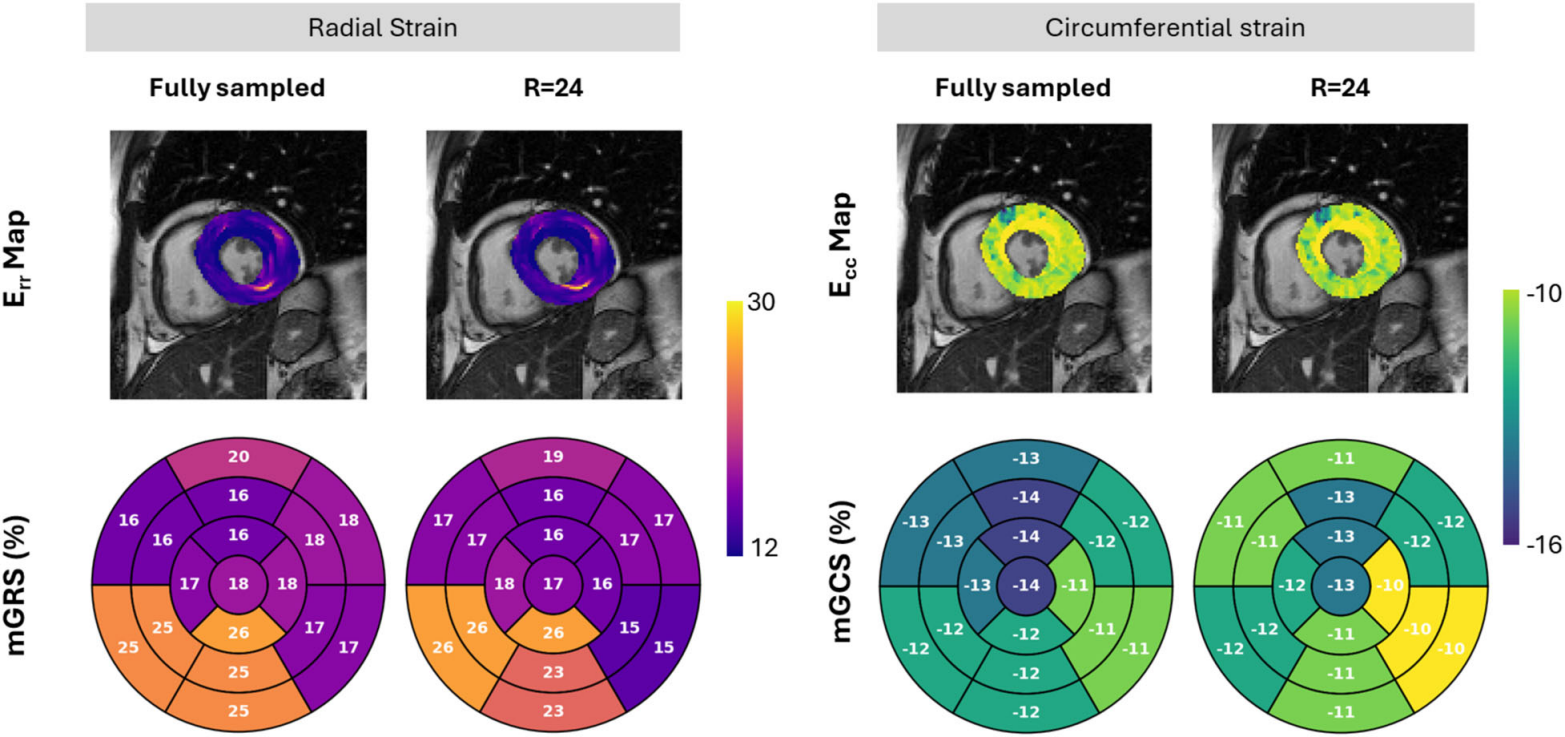
Strain map distributions of the radial $E_{rr}$ and circumferential $E_{cc}$ strain, overlaid on the systolic frame for a patient with antisynthetase syndrome. Corresponding bull's‐eye plots illustrate the mean global radial strain (mGRS) and mean global circumferential strain (mGCS). Visual comparison between fully sampled and R=24 accelerated cases demonstrates the robustness of the proposed method in computing strain metrics under highly accelerated conditions.

Additionally, mean global radial (mGRS) and circumferential (mGCS) strain measurements derived from fully sampled data revealed strong agreement (Figure S6) with those obtained from accelerated data (mGRS: r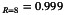, r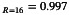, r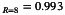, p‐value = 0.994, mGCS: r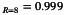, r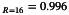, r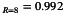, p‐value = 0.991). We assessed regional mean radial and circumferential systolic strains in a patient with antisynthetase syndrome for fully sampled and R=24 accelerated data (Figure 9). The resulting radial and circumferential strain maps and bull's‐eye plots demonstrated comparable strain magnitudes and distributions. Minimal discrepancies in segmental strain values showcased robust strain estimation under high acceleration.

### 3.3 Out‐of‐Distribution Data

Post‐contrast (Figure S7) and radial data (Figure S8), were successfully reconstructed and segmented. In each case, cardiac motion was reliably captured across varying accelerations. However, at extreme undersampling (R>24), image quality degraded, with local motion distortions, and reduced segmentation accuracy (Figure S9).

MOPNet also preserved clear ventricular boundaries and realistic cardiac dynamics in CMRxRecon scans despite spatial resolution differences. While manual segmentation ground truth is unavailable, the predicted segmentation remained anatomically meaningful without collapse, though minor boundary deviations were noted (Figure S10). Additionally, the motion estimates are consistent with the underlying motion relaxation dynamics across previously unseen long‐axis views (Figure S11).

### 3.4 Ablation Study

**FIGURE 10:**
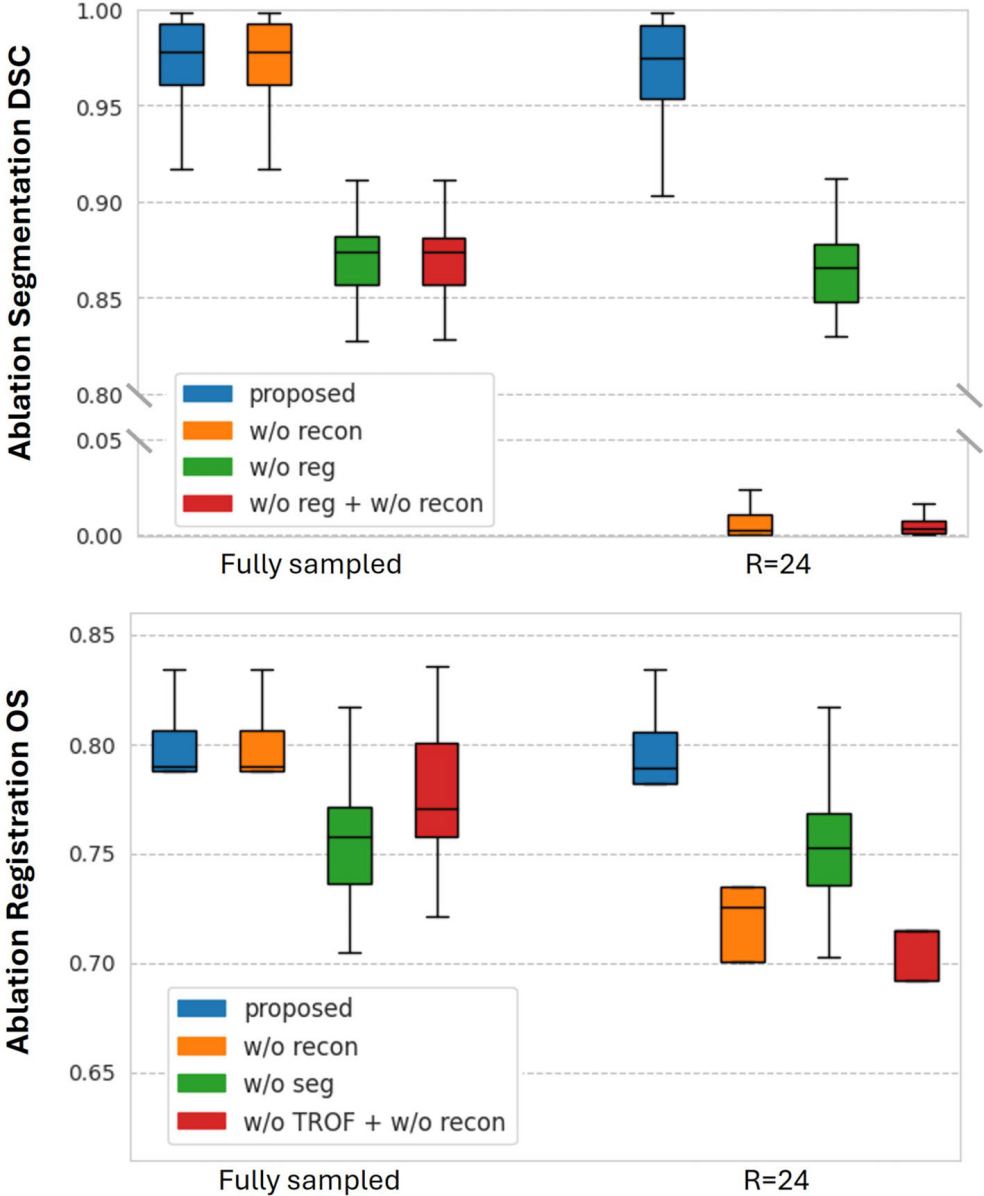
Boxplots (horizontal line: median, box: 25th/75th percentile, standard deviation: whiskers) of the mean DSC and OS of the right ventricular and left ventricular blood pools, derived from the segmentation and registration networks in the ablation study. Metrics are shown for the fully sampled and R=24 accelerated cases, obtained through fourfold cross‐validation.

The ablation study (Figure 10) demonstrated that the proposed training and framework design consistently achieved the highest mean segmentation DSC and registration OS. The reconstruction module significantly improved both metrics at the R=24 accelerated case. Complementary qualitative comparison (Figure S12) shows that motion compensation improves image fidelity, with enhanced structural sharpness, better anatomical consistency, and reduced error residuals. Removing image registration significantly reduced the segmentation DSC. Likewise, omitting joint training reduced the registration OS. This demonstrates that joint training yielded superior performance compared to standalone approaches. The registration and reconstruction modules contributed to the consistent segmentation performance at R=24, with DSC comparable to the fully sampled case. Additionally, multi‐frame image registration exhibited higher mean OS and reduced variance compared to pairwise registration, indicated by the narrower interquartile range. This was particularly evident in the fully sampled case, where aliasing artifacts were absent.

## 4 Discussion

This study introduces a novel deep learning‐based framework for ventricular function assessment in fully sampled and accelerated cine MRI. The existing methods primarily address image registration, reconstruction, and segmentation separately, despite evidence suggesting mutual benefits from a combined approach [23, 33, 36]. Furthermore, prior single breath‐hold acquisition techniques for full left ventricular coverage [6, 54, 55] primarily prioritize acquisition speed, overlooking downstream tasks like motion estimation and segmentation at high accelerations. In contrast, we integrate these interdependent tasks into a unified framework to leverage their synergy for improved performance and ensure robustness to high acceleration.

Current MRI assessments for ventricular function are often slow and prone to errors, hindering efficient cardiac condition screening. These limitations can be attributed to various factors, including inter‐observer bias [56], inconsistent labeling protocols [57], and the prolonged scan time. Our findings demonstrate the viability of a unified registration, reconstruction, and segmentation strategy to address these challenges.

Central to our approach is MOPNet, a novel groupwise image registration model that explicitly encodes temporal features and produces reliable motion estimates from accelerated data. These motion predictions simultaneously enhance the image reconstruction and improve the segmentation performance. The integrated motion‐compensated reconstruction, in turn, produces high‐quality images, mitigating aliasing artifacts caused by high acceleration. These aliasing‐free images can be leveraged to improve registration and generate more suitable inputs for accurate image segmentation. The segmentation network provides feedback to the registration process, offering anatomical guidance for accurate motion mapping.

The proposed method demonstrated superior performance compared to other deep‐learning segmentation models in segmentation and volumetric measurements. The competing methods included previous joint registration and segmentation frameworks (MotionNet [32], DeepAtlas [33]) and state‐of‐the‐art segmentation networks (nnUNet [12], FCT [13]). These networks encountered difficulties segmenting apical and basal slices, a common challenge in cardiac image segmentation [58]. We addressed this limitation effectively while maintaining the computational efficiency of a simple convolutional network. The ablation study confirmed that the joint training strategy was key to this improvement, consistent with findings from previous works [32, 33]. The pseudo‐labels generated through image registration provided additional supervision that improved the learning from the limited available annotations. Moreover, the resulting volumetric measures remained comparable to expert readings, even at accelerations up to R=24. Therefore, our strategy enables reliable segmentation and volumetric measures extraction, even without access to fully sampled data.

MOPNet achieved precise motion estimation due to three primary factors: iterative refinement, self‐attention priors, demonstrated in GMA‐RAFT [19], and rich contextual features derived from multiple temporal frames, as evidenced in the ablation study. This combination of spatial, temporal, global, and local features helps resolve ambiguities and provides reliable motion estimates in the presence of undersampling artifacts. Explicit temporal feature extraction offered a significant advantage over the common pairwise learning strategy [11, 19], which is in line with previous findings [23, 24]. Learning temporal motion features enables capturing complex motion dynamics, which pairwise comparisons may fail to detect.

We also demonstrated consistent global and regional strain measurements across different accelerations. A direct comparison with existing literature is challenging, as reported strain ranges vary significantly depending on the imaging modality and computation technique [59]. Nonetheless, the results provide initial proof‐of‐value, highlighting the potential for developing more efficient strain analysis workflows. Comprehensive benchmarking against established clinical standards will be required to validate diagnostic accuracy.

We acknowledge several limitations of this work. The motion‐compensated kt‐SLR reconstruction is computationally intensive (15 min/subject), due to iterative optimization, whereas registration (= 1 min) and segmentation (= 0.2 s) contribute minimally to the runtime. This overhead arises as a trade‐off to capture long‐range temporal correlations and coherently suppress aliasing at high accelerations (R≥16). Sliding‐window techniques [8, 60, 61] offer an efficient alternative, but remain prone to temporal blurring, inaccurate depiction of rapid dynamics, and propagation of motion errors [19, 23, 62], limiting their efficacy for functional analysis at our target high accelerations. Future integration of the global and sliding‐window paradigms via deep learning could optimize the accuracy‐efficiency balance [6, 7, 8, 9]. Shared feature representations across modules may further reduce redundancy and improve overall performance [63, 64].

Our current motion estimation relies on brightness constancy and smoothness assumptions, which limit performance in cases of through‐plane motion, such as basal slices or myocardial twisting. Failures in registration can also generate inaccurate pseudo‐labels, compromising segmentation. Reliability can be improved through consistency checks across frames [65], 3D motion modeling [11], predictive uncertainty estimation [66], or fusing predictions from overlapping windows and multiple strides.

Initial results suggest promising generalization across acquisition settings and views. MOPNet makes no explicit assumptions on cardiac morphology, enabling generalization to unseen long‐axis views, radial acquisitions, and post‐contrast images. Nonetheless, segmentation remains sensitive to task‐ or domain‐specific factors. Performance degrades at accelerations above R=24, on out‐of‐domain data (e.g., high‐resolution CMRxRecon) and may also be challenged by complex anatomies (e.g., congenital heart disease, myocardial scarring, or abnormal wall motion). Unreliable predictions can be identified via automated quality‐control [67, 68] or uncertainty maps [69, 70]. Possible mitigation strategies include using anatomical shape priors [71], targeted fine‐tuning, or the generation of synthetic normal and pathological deformations to reduce dependence on manual annotations [72].

Another major consideration for broader applicability is the performance in patients with arrhythmia, where irregular heartbeats disrupt temporal consistency. In such cases, complementary self‐gating modules [73] or real‐time imaging and motion estimation [74, 75] could be integrated to preserve reconstruction fidelity and thereby enable reliable functional assessment.

Future work will include systematic and prospective validation of the different tasks in diverse populations and across vendors, institutions, and imaging protocols, performed on short‐ and long‐axis views. We will also benchmark the quantitative analysis against independent reference standards, including motion‐tracking methods (e.g., tagging, DENSE) and commercial strain analysis tools. Achieving accurate strain analysis in future studies will require ensuring high temporal resolution [76, 77]. Extension to 3T acquisitions will additionally require addressing B0/B1+ inhomogeneity artifacts through artifact‐robust pre‐processing or domain‐adaptive fine‐tuning [78].

## 5 Conclusion

We presented an automated framework integrating multi‐frame image registration, motion‐compensated reconstruction, and segmentation to enable comprehensive cardiac functional assessment. By combining these techniques, the reliability of each task is enhanced across different acceleration rates, ranging from fully sampled to highly accelerated data of up to R=24. The proposed method has the potential to increase the efficiency of cardiac MRI workflows by providing a versatile set of operator‐independent tasks. These results hold promise for the comprehensive extraction of relevant clinical parameters, such as volumetric and myocardial strain measurements, within a single breath‐hold in future prospective studies.

## Supporting information




**Data S1**: Supporting Information.

## Data Availability

The source code is made publicly available on https://github.com/lab‐midas/MOPNet.

## References

[1] J. P. Ridgway , “Cardiovascular Magnetic Resonance Physics for Clinicians: Part I,” Journal of Cardiovascular Magnetic Resonance 12, no. 1 (2010): 71. 21118531 10.1186/1532-429X-12-71 PMC3016368

[2] M. A. Konstam and F. M. Abboud , “Ejection Fraction: Misunderstood and Overrated (Changing the Paradigm in Categorizing Heart Failure),” Circulation 135, no. 8 (2017): 717–719. 28223323 10.1161/CIRCULATIONAHA.116.025795 PMC5325053

[3] C. Piet , O. A. M. Salem , P. Gianni , P. P. Sengupta , and E. Nagel , “Tissue Tracking Technology for Assessing Cardiac Mechanics: Principles, Normal Values, and Clinical Applications,” JACC: Cardiovascular Imaging 8, no. 12 (2015): 1444–1460. 26699113 10.1016/j.jcmg.2015.11.001

[4] B. P. Halliday , R. Senior , and D. J. Pennell , “Assessing Left Ventricular Systolic Function: From Ejection Fraction to Strain Analysis,” European Heart Journal 42, no. 7 (2021): 789–797. 32974648 10.1093/eurheartj/ehaa587

[5] M. Safari , Z. Eidex , C.‐W. Chang , R. L. J. Qiu , and X. Yang , “Advancing MRI Reconstruction: A Systematic Review of Deep Learning and Compressed Sensing Integration,” (2025), arXiv preprint arXiv:2501.14158.

[6] S. Xu , K. Hammernik , A. Lingg , et al., “Attention Incorporated Network for Sharing Low‐Rank, Image and k‐Space Information During MR Image Reconstruction to Achieve Single Breath‐Hold Cardiac Cine Imaging,” Computerized Medical Imaging and Graphics 120 (2025): 102475. 39808868 10.1016/j.compmedimag.2024.102475

[7] T. Küstner , N. Fuin , K. Hammernik , et al., “CINENet: Deep Learning‐Based 3D Cardiac CINE MRI Reconstruction With Multi‐Coil Complex‐Valued 4D Spatio‐Temporal Convolutions,” Scientific Reports 10, no. 1 (2020): 13710. 32792507 10.1038/s41598-020-70551-8 PMC7426830

[8] C. M. Sandino , P. Lai , S. S. Vasanawala , and J. Y. Cheng , “Accelerating Cardiac Cine MRI Using a Deep Learning‐Based ESPIRiT Reconstruction,” Magnetic Resonance in Medicine 85, no. 1 (2021): 152–167. 32697891 10.1002/mrm.28420 PMC7722220

[9] H. Kerstin and A. Mehmet , “Artificial Intelligence for Image Enhancement and Reconstruction in Magnetic Resonance Imaging,” in Artificial Intelligence in Cardiothoracic Imaging (Springer, 2022), 125–138.

[10] V. Spieker , H. Eichhorn , K. Hammernik , et al., “Deep Learning for Retrospective Motion Correction in MRI: A Comprehensive Review,” IEEE Transactions on Medical Imaging 43, no. 2 (2023): 846–859. 10.1109/TMI.2023.3323215 37831582

[11] B. Guha , Z. Amy , R. Sabuncu Mert , G. John , and V. Dalca Adrian , “Voxelmorph: A Learning Framework for Deformable Medical Image Registration,” IEEE Transactions on Medical Imaging 38, no. 8 (2019): 1788–1800. 10.1109/TMI.2019.2897538 30716034

[12] F. Isensee , P. F. Jaeger , S. A. A. Kohl , J. Petersen , and K. H. Maier‐Hein , “nnU‐Net: A Self‐Configuring Method for Deep Learning‐Based Biomedical Image Segmentation,” Nature Methods 18, no. 2 (2021): 203–211. 33288961 10.1038/s41592-020-01008-z

[13] A. Tragakis , C. Kaul , R. Murray‐Smith , and D. Husmeier , “The Fully Convolutional Transformer for Medical Image Segmentation,” in Proceedings of the IEEE/CVF Winter Conference on Applications of Computer Vision (IEEE, 2023), 3660–3669.

[14] A. N. Bhuva , W. Bai , C. Lau , et al., “A Multicenter, Scan‐Rescan, Human and Machine Learning CMR Study to Test Generalizability and Precision in Imaging Biomarker Analysis,” Circulation: Cardiovascular Imaging 12, no. 10 (2019): e009214. 31547689 10.1161/CIRCIMAGING.119.009214

[15] Q. Vo Ha , T. H. Marwick , and K. Negishi , “MRI‐Derived Myocardial Strain Measures in Normal Subjects,” JACC: Cardiovascular Imaging 11, no. 2 Part 1 (2018): 196–205. 28528164 10.1016/j.jcmg.2016.12.025

[16] M. A. Morales , D. Izquierdo‐Garcia , I. Aganj , J. Kalpathy‐Cramer , B. R. Rosen , and C. Catana , “Implementation and Validation of a Three‐Dimensional Cardiac Motion Estimation Network,” Radiology: Artificial Intelligence 1, no. 4 (2019): e180080. 32076659 10.1148/ryai.2019180080 PMC6677286

[17] R. R. Upendra , B. J. Wentz , S. M. Shontz , and C. A. Linte , “A Convolutional Neural Network‐Based Deformable Image Registration Method for Cardiac Motion Estimation From Cine Cardiac MR Images,” in Proceedings of the 2020 Computing in Cardiology (IEEE, 2020), 1–4. 10.22489/CinC.2020.204 PMC8168986 34079839

[18] H. Yu , X. Chen , H. Shi , T. Chen , T. S. Huang , and S. Sun , “Motion Pyramid Networks for Accurate and Efficient Cardiac Motion Estimation,” in Medical Image Computing and Computer Assisted Intervention–MICCAI 2020: 23rd International Conference, Lima, Peru, October 4–8, 2020, Proceedings, Part VI, vol. 23 (Springer, 2020), 436–446.

[19] A. Ghoul , J. Pan , A. Lingg , et al., “Attention‐Aware Non‐Rigid Image Registration for Accelerated MR Imaging,” IEEE Transactions on Medical Imaging 43 (2024): 3013–3026. 39088484 10.1109/TMI.2024.3385024

[20] M. Ye , M. Kanski , and D. Yang , “Deeptag: An Unsupervised Deep Learning Method for Motion Tracking on Cardiac Tagging Magnetic Resonance Images,” in Proceedings of the IEEE/CVF Conference on Computer Vision and Pattern Recognition (IEEE, 2021), 7261–7271.

[21] M. De Craene , C. Tobon‐Gomez , C. Butakoff , et al., “Temporal Diffeomorphic Free Form Deformation (TDFFD) Applied to Motion and Deformation Quantification of Tagged MRI Sequences,” in Statistical Atlases and Computational Models of the Heart (Springer, 2012), 68–77.

[22] E. Martín‐González , T. Sevilla , A. Revilla‐Orodea , P. Higuera , and C. Alberola‐López , “Groupwise Non‐Rigid Registration With Deep Learning: An Affordable Solution Applied to 2D Cardiac Cine MRI Reconstruction,” Entropy 22, no. 6 (2020): 687. 33286459 10.3390/e22060687 PMC7517224

[23] J. Pan , D. Rueckert , T. Küstner , and K. Hammernik , “Learning‐Based and Unrolled Motion‐Compensated Reconstruction for Cardiac MR CINE Imaging,” in International Conference on Medical Image Computing and Computer‐Assisted Intervention (Springer, 2022), 686–696.

[24] P. Qian , J. Yang , P. Lió , et al., “Joint Group‐Wise Motion Estimation and Segmentation of Cardiac Cine MR Images Using Recurrent U‐Net,” in Annual Conference on Medical Image Understanding and Analysis (Springer, 2022), 65–74.

[25] X. Shi , Z. Huang , W. Bian , et al., “Videoflow: Exploiting Temporal Cues for Multi‐Frame Optical Flow Estimation,” in Proceedings of the IEEE/CVF International Conference on Computer Vision (IEEE, 2023), 12469–12480.

[26] M. Usman , D. Atkinson , F. Odille , et al., “Motion Corrected Compressed Sensing for Free‐Breathing Dynamic Cardiac MRI,” Magnetic Resonance in Medicine 70, no. 2 (2013): 504–516. 22899104 10.1002/mrm.24463

[27] A. Budai , F. I. Suhai , K. Csorba , et al., “Fully Automatic Segmentation of Right and Left Ventricle on Short‐Axis Cardiac MRI Images,” Computerized Medical Imaging and Graphics 85 (2020): 101786. 32866695 10.1016/j.compmedimag.2020.101786

[28] V. M. Campello , G. Polyxeni , C. Izquierdo , et al., “Multi‐Centre, Multi‐Vendor and Multi‐Disease Cardiac Segmentation: The M&Ms Challenge,” IEEE Transactions on Medical Imaging 40, no. 12 (2021): 3543–3554. 34138702 10.1109/TMI.2021.3090082

[29] O. Bernard , A. Lalande , C. Zotti , et al., “Deep Learning Techniques for Automatic MRI Cardiac Multi‐Structures Segmentation and Diagnosis: Is the Problem Solved?,” IEEE Transactions on Medical Imaging 37, no. 11 (2018): 2514–2525. 29994302 10.1109/TMI.2018.2837502

[30] M. A. Morales , M. Boomen , C. Nguyen , et al., “DeepStrain: A Deep Learning Workflow for the Automated Characterization of Cardiac Mechanics,” Frontiers in Cardiovascular Medicine 8 (2021): 730316. 34540923 10.3389/fcvm.2021.730316 PMC8446607

[31] C. V. Graves , M. F. S. Rebelo , R. A. Moreno , et al., “Siamese Pyramidal Deep Learning Network for Strain Estimation in 3D Cardiac Cine‐MR,” Computerized Medical Imaging and Graphics 108 (2023): 102283. 37562136 10.1016/j.compmedimag.2023.102283

[32] Q. Chen , B. Wenjia , S. Jo , et al., “Joint Learning of Motion Estimation and Segmentation for Cardiac MR Image Sequences,” in Medical Image Computing and Computer Assisted Intervention–MICCAI 2018: 21st International Conference, Granada, Spain, September 16–20, 2018, Proceedings, Part II, vol. 11 (Springer, 2018), 472–480.

[33] N. M. Xu Zhenlin , “DeepAtlas: Joint Semi‐Supervised Learning of Image Registration and Segmentation,” in Medical Image Computing and Computer Assisted Intervention–MICCAI 2019: 22nd International Conference, Shenzhen, China, October 13–17, 2019, Proceedings, Part II, vol. 22 (Springer, 2019), 420–429. 10.1007/978-3-030-32245-8_47 PMC11378322 39247524

[34] J. Pan , D. Rueckert , T. Küstner , and K. Hammernik , “Efficient Image Registration Network for Non‐Rigid Cardiac Motion Estimation,” in Machine Learning for Medical Image Reconstruction: 4th International Workshop, MLMIR 2021, Held in Conjunction With MICCAI 2021, Strasbourg, France, October 1, 2021, Proceedings 4 (Springer, 2021), 14–24.

[35] J. Yang , T. Küstner , P. Hu , P. Liò , and H. Qi , “End‐To‐End Deep Learning of Non‐Rigid Groupwise Registration and Reconstruction of Dynamic MRI,” Frontiers in Cardiovascular Medicine 9 (2022): 880186. 35571217 10.3389/fcvm.2022.880186 PMC9095964

[36] P. Qian , Z. Zhou , P. Hu , and H. Qi , “Unified Deep Learning for Simultaneous Cardiac Cine MRI Reconstruction, Motion Estimation and Segmentation,” in Proceedings of the 2024 IEEE International Symposium on Biomedical Imaging (ISBI) (IEEE, 2024), 1–4.

[37] S. G. Lingala , Y. Hu , E. DiBella , and M. Jacob , “Accelerated Dynamic MRI Exploiting Sparsity and Low‐Rank Structure: Kt SLR,” IEEE Transactions on Medical Imaging 30, no. 5 (2011): 1042–1054. 21292593 10.1109/TMI.2010.2100850 PMC3707502

[38] O. Ronneberger , P. Fischer , and T. Brox , “U‐Net: Convolutional Networks for Biomedical Image Segmentation,” in Medical Image Computing and Computer‐Assisted Intervention–MICCAI 2015: 18th International Conference, Munich, Germany, October 5–9, 2015, Proceedings, Part III 18 (Springer, 2015), 234–241.

[39] J. Shihao , C. Dylan , L. Yao , L. Hongdong , and H. Richard , “Learning to Estimate Hidden Motions With Global Motion Aggregation,” in Proceedings of the IEEE/CVF International Conference on Computer Vision (IEEE, 2021), 9772–9781.

[40] Z. Teed and J. Deng , “Raft: Recurrent All‐Pairs Field Transforms for Optical Flow,” in Computer Vision–ECCV 2020: 16th European Conference, Glasgow, UK, August 23–28, 2020, Proceedings, Part II 16 (Springer, 2020), 402–419.

[41] S. Sun , Y. Chen , Y. Zhu , G. Guo , and G. Li , “Skflow: Learning Optical Flow With Super Kernels,” Advances in Neural Information Processing Systems 35 (2022): 11313–11326.

[42] P. G. Batchelor , D. Atkinson , P. Irarrazaval , D. L. G. Hill , J. Hajnal , and D. Larkman , “Matrix Description of General Motion Correction Applied to Multishot Images,” Magnetic Resonance in Medicine 54, no. 5 (2005): 1273–1280. 16155887 10.1002/mrm.20656

[43] D. C. M. Oubel Estanislao and A. O. Hero , “Cardiac Motion Estimation by Joint Alignment of Tagged MRI Sequences,” Medical Image Analysis 16, no. 1 (2012): 339–350. 22000567 10.1016/j.media.2011.09.001 PMC4401871

[44] T. Belytschko , W. K. Liu , B. Moran , and K. Elkhodary , Nonlinear Finite Elements for Continua and Structures (John wiley & sons, 2014).

[45] M. D. Cerqueira , N. J. Weissman , V. Dilsizian , et al., “Standardized Myocardial Segmentation and Nomenclature for Tomographic Imaging of the Heart: A Statement for Healthcare Professionals From the Cardiac Imaging Committee of the Council on Clinical Cardiology of the American Heart Association,” Circulation 105, no. 4 (2002): 539–542. 11815441 10.1161/hc0402.102975

[46] E. Heiberg , J. Sjögren , M. Ugander , M. Carlsson , H. Engblom , and H. Arheden , “Design and Validation of Segment‐Freely Available Software for Cardiovascular Image Analysis,” BMC Medical Imaging 10 (2010): 1–13. 20064248 10.1186/1471-2342-10-1 PMC2822815

[47] R. Ahmad , H. Xue , S. Giri , Y. Ding , J. Craft , and O. P. Simonetti , “Variable Density Incoherent Spatiotemporal Acquisition (VISTA) for Highly Accelerated Cardiac MRI,” Magnetic Resonance in Medicine 74, no. 5 (2015): 1266–1278. 25385540 10.1002/mrm.25507 PMC4556611

[48] M. Uecker , P. Lai , M. J. Murphy , et al., “ESPIRiT—An Eigenvalue Approach to Autocalibrating Parallel MRI: Where SENSE Meets GRAPPA,” Magnetic Resonance in Medicine 71, no. 3 (2014): 990–1001. 23649942 10.1002/mrm.24751 PMC4142121

[49] S. Winkelmann , T. Schaeffter , T. Koehler , H. Eggers , and O. Doessel , “An Optimal Radial Profile Order Based on the Golden Ratio for Time‐Resolved MRI,” IEEE Transactions on Medical Imaging 26, no. 1 (2006): 68–76. 10.1109/TMI.2006.885337 17243585

[50] J. Lyu , C. Qin , S. Wang , et al., “The State‐Of‐The‐Art in Cardiac Mri Reconstruction: Results of the Cmrxrecon Challenge in Miccai 2023,” Medical Image Analysis 101 (2025): 103485. 39946779 10.1016/j.media.2025.103485

[51] I. Loshchilov and F. Hutter , “Decoupled Weight Decay Regularization,” (2017), arXiv preprint arXiv:1711.05101.

[52] I. Loshchilov and F. Hutter , “Sgdr: Stochastic Gradient Descent With Warm Restarts,” (2016), arXiv preprint arXiv:1608.03983.

[53] S. Baker , D. Scharstein , J. P. Lewis , S. Roth , M. J. Black , and R. Szeliski , “A Database and Evaluation Methodology for Optical Flow,” International Journal of Computer Vision 92 (2011): 1–31.

[54] T. Kido , T. Kido , M. Nakamura , et al., “Compressed Sensing Real‐Time Cine Cardiovascular Magnetic Resonance: Accurate Assessment of Left Ventricular Function in a Single‐Breath‐Hold,” Journal of Cardiovascular Magnetic Resonance 18, no. 1 (2016): 50. 27553656 10.1186/s12968-016-0271-0 PMC4995641

[55] D. Kim , J. Coll‐Font , R. A. Lobos , et al., “Single Breath‐Hold CINE Imaging With Combined Simultaneous Multislice and Region‐Optimized Virtual Coils,” Magnetic Resonance in Medicine 90, no. 1 (2023): 222–230. 36864561 10.1002/mrm.29620 PMC10315014

[56] A. Suinesiaputra , B. R. Cowan , A. O. Al‐Agamy , et al., “A Collaborative Resource to Build Consensus for Automated Left Ventricular Segmentation of Cardiac MR Images,” Medical Image Analysis 18, no. 1 (2014): 50–62. 24091241 10.1016/j.media.2013.09.001 PMC3840080

[57] Z. Qiao , H. Delingette , N. Duchateau , and N. Ayache , “3‐D Consistent and Robust Segmentation of Cardiac Images by Deep Learning With Spatial Propagation,” IEEE Transactions on Medical Imaging 37, no. 9 (2018): 2137–2148. 29994087 10.1109/TMI.2018.2820742

[58] K. Mahendra , K. V. Alex , and K. Ganapathy , “Fully Convolutional Multi‐Scale Residual DenseNets for Cardiac Segmentation and Automated Cardiac Diagnosis Using Ensemble of Classifiers,” Medical Image Analysis 51 (2019): 21–45. 30390512 10.1016/j.media.2018.10.004

[59] M. S. Amzulescu , M. De Craene , H. Langet , et al., “Myocardial Strain Imaging: Review of General Principles, Validation, and Sources of Discrepancies,” European Heart Journal 20, no. 6 (2019): 605–619. 30903139 10.1093/ehjci/jez041 PMC6529912

[60] M. S. Hansen , T. S. Sørensen , A. E. Arai , and P. Kellman , “Retrospective Reconstruction of High Temporal Resolution Cine Images From Real‐Time MRI Using Iterative Motion Correction,” Magnetic Resonance in Medicine 68, no. 3 (2012): 741–750. 22190255 10.1002/mrm.23284 PMC3311753

[61] S. Jung Hong , S. Kyunghyun , S. Nayak Krishna , K. E. Yeop , and Y. J. Chul , “K‐t FOCUSS: A General Compressed Sensing Framework for High Resolution Dynamic MRI,” Magnetic Resonance in Medicine 61, no. 1 (2009): 103–116. 19097216 10.1002/mrm.21757

[62] J. Tsao , P. Boesiger , and K. P. Pruessmann , “K‐t BLAST and k‐t SENSE: Dynamic MRI With High Frame Rate Exploiting Spatiotemporal Correlations,” Magnetic Resonance in Medicine 50, no. 5 (2003): 1031–1042. 14587014 10.1002/mrm.10611

[63] Q. Chang , Z. Yan , M. Zhou , et al., “Deeprecon: Joint 2d Cardiac Segmentation and 3d Volume Reconstruction via a Structure‐Specific Generative Method,” in International Conference on Medical Image Computing and Computer‐Assisted Intervention (Springer, 2022), 567–577.

[64] T. Wech , O. Schad , S. Sauer , et al., “Joint Image Reconstruction and Segmentation of Real‐Time Cardiovascular Magnetic Resonance Imaging in Free‐Breathing Using a Model Based on Disentangled Representation Learning,” Journal of Cardiovascular Magnetic Resonance 27, no. 1 (2025): 101844. 39864743 10.1016/j.jocmr.2025.101844 PMC11874730

[65] J. Jeong Jisoo , H. Cai Hong , R. Garrepalli Risheek , J. M. Lin Jamie Menjay , M. Hayat Munawar , and F. Porikli Fatih , “Ocai: Improving Optical Flow Estimation by Occlusion and Consistency Aware Interpolation,” in Proceedings of the IEEE/CVF Conference on Computer Vision and Pattern Recognition (IEEE, 2024), 19352–19362.

[66] E. Ilg , O. Cicek , S. Galesso , et al., “Uncertainty Estimates and Multi‐Hypotheses Networks for Optical Flow,” in Proceedings of the European Conference on Computer Vision (ECCV) (Springer 2018), 652–667.

[67] S. Bohlender , I. Oksuz , and A. Mukhopadhyay , “A Survey on Shape‐Constraint Deep Learning for Medical Image Segmentation,” IEEE Reviews in Biomedical Engineering 16 (2021): 225–240. 10.1109/RBME.2021.3136343 34919522

[68] G. Simantiris and G. Tziritas , “Cardiac MRI Segmentation With a Dilated CNN Incorporating Domain‐Specific Constraints,” IEEE Journal of Selected Topics in Signal Processing 14, no. 6 (2020): 1235–1243.

[69] X. Alba , K. Lekadir , M. Pereanez , P. Medrano‐Gracia , A. A. Young , and A. F. Frangi , “Automatic Initialization and Quality Control of Large‐Scale Cardiac MRI Segmentations,” Medical Image Analysis 43 (2018): 129–141. 29073531 10.1016/j.media.2017.10.001

[70] J. Sander , B. D. Vos , and I. Išgum , “Automatic Segmentation With Detection of Local Segmentation Failures in Cardiac MRI,” Scientific Reports 10, no. 1 (2020): 21769. 33303782 10.1038/s41598-020-77733-4 PMC7729401

[71] L. van Harten , R. L. M. Van Herten , J. Stoker , and I. Isgum , “Deformable Image Registration With Geometry‐Informed Implicit Neural Representations,” in Medical Imaging With Deep Learning (PMLR, 2024), 730–742. 10.1109/TMI.2023.3321425 37782589

[72] J. Krebs , H. Delingette , B. Mailhé , N. Ayache , and T. Mansi , “Learning a Probabilistic Model for Diffeomorphic Registration,” IEEE Transactions on Medical Imaging 38, no. 9 (2019): 2165–2176. 30716033 10.1109/TMI.2019.2897112

[73] F. Contijoch , S. K. Iyer , J. J. Pilla , et al., “Self‐Gated MRI of Multiple Beat Morphologies in the Presence of Arrhythmias,” Magnetic Resonance in Medicine 78, no. 2 (2017): 678–688. 27579717 10.1002/mrm.26381 PMC5332534

[74] L. Feng , M. B. Srichai , R. P. Lim , et al., “Highly Accelerated Real‐Time Cardiac Cine MRI Using k–t SPARSE‐SENSE,” Magnetic Resonance in Medicine 70, no. 1 (2013): 64–74. 22887290 10.1002/mrm.24440 PMC3504620

[75] A. Ghoul , K. Hammernik , A. Lingg , et al., “Highly Efficient Non‐Rigid Registration in k‐Space With Application to Cardiac Magnetic Resonance Imaging,” (2024), arXiv preprint arXiv:2410.18834.

[76] J. Schmidt‐Rimpler , S. J. Backhaus , F. P. Hartmann , et al., “Impact of Temporal and Spatial Resolution on Atrial Feature Tracking Cardiovascular Magnetic Resonance Imaging,” International Journal of Cardiology 396 (2024): 131563. 37926379 10.1016/j.ijcard.2023.131563

[77] W. Yang , H. Li , J. He , et al., “Left Ventricular Strain Measurements Derived From MR Feature Tracking: A Head‐To‐Head Comparison of a Higher Temporal Resolution Method With a Conventional Method,” Journal of Magnetic Resonance Imaging 56, no. 3 (2022): 801–811. 35005810 10.1002/jmri.28053

[78] S. Ram , S. Navdeep , and L. Kaur , “Deep Learning Methods for 3D Magnetic Resonance Image Denoising, Bias Field and Motion Artifact Correction: A Comprehensive Review,” Physics in Medicine and Biology 96 (2024): 23TR01. 10.1088/1361-6560/ad94c7 39569887

