# Supplementary material for: Multi‐Frame Image Registration for Automated Ventricular Function Assessment in Single Breath‐Hold Cine MRI Using Limited Labels
Source: Magn Reson Med. 2025 Oct 18;95(3):1762–77. doi: 10.1002/mrm.70137 (PMC12746400; doi:10.1002/mrm.70137)
Supplement: Supplementary file 1 — Data S1: Supporting Information. [file MRM-95-1762-s001.pdf]

Supporting Information

Multi-Frame Image Registration for Automated Ventricular Function Assessment in Single Breath-Hold Cine MRI Using Limited Labels

Aya Ghoul[1], Paxstan Cassal Paulson[2], Kerstin Hammernik[3], Andreas Lingg[4], Patrick Krumm[4], Daniel Rueckert[3,5,6], Sergios Gatidis[1,7], and Thomas Küstner[1]

[1]Medical Image and Data Analysis (MIDAS.lab), Department of Diagnostic and Interventional Radiology, University Hospital of Tuebingen, Germany
[2]Institute of Signal Processing and System Theory, University of Stuttgart, Stuttgart, Germany
[3]School of Computation, Information and Technology, Technical University of Munich, Germany
[4]Department of Diagnostic and Interventional Radiology, University Hospital of Tuebingen, Germany
[5]Klinikum Rechts der Isar, Technical University of Munich, Germany
[6]Department of Computing, Imperial College London, UK
[7]Department of Radiology, Stanford University, USA

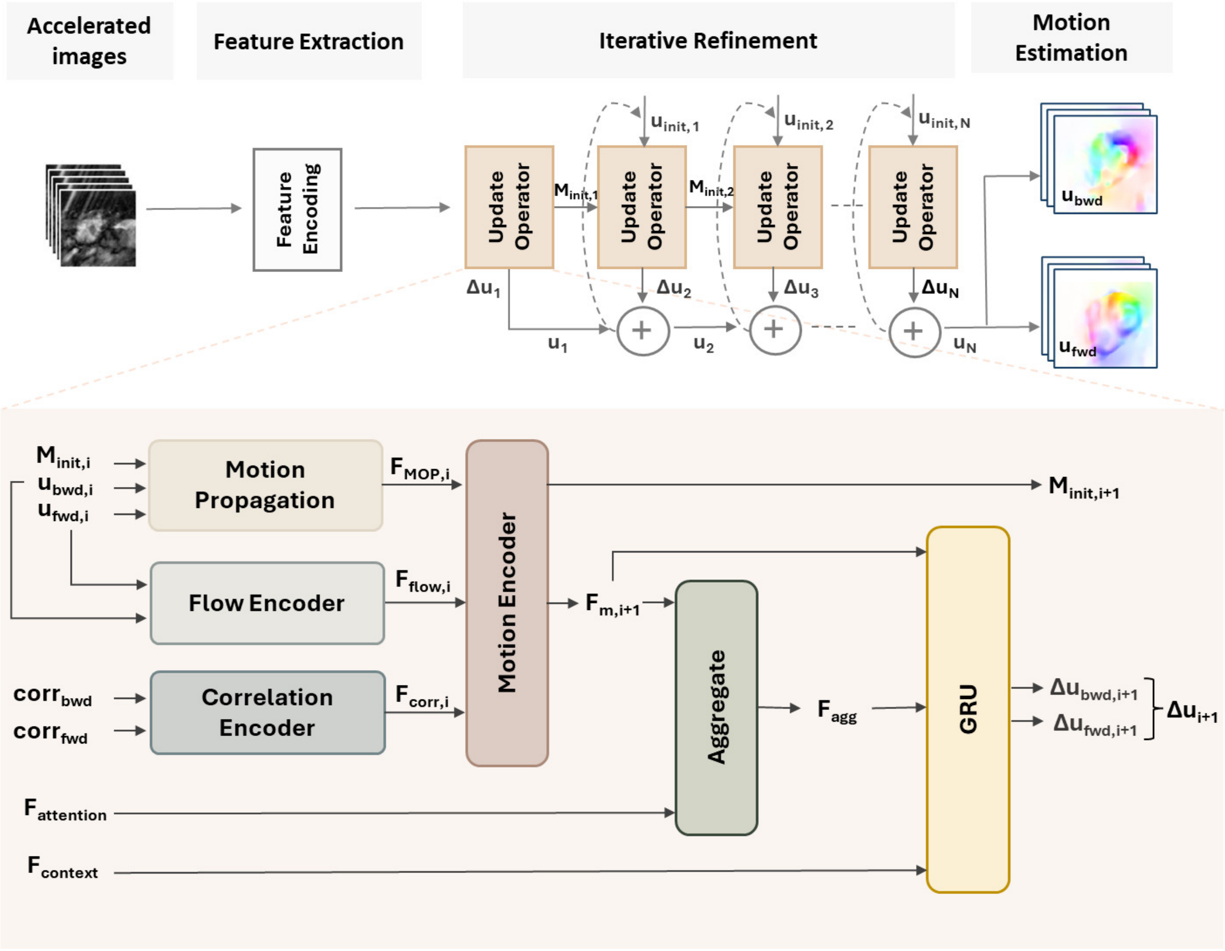

Figure S1: The Update Operator of the motion propagation network (MOPNet) overview. The Motion Propagation (MOP) Module explicitly leverages temporal context by maintaining and updating hidden motion states M_{init} of the input image triplets. Long-range temporal information is progressively incorporated. At each iteration, the update module decodes current flow estimations ($u_{bwd,i}$ and $u_{fwd,i}$), correlation (F_{corr}), MOP (F_{MOP}), attention ($F_{attention}$) and context ($F_{context}$) features using modified gated recurrent units (GRUs) to output bidirectional residual flows ($\Delta u_{bwd,i}$, $\Delta u_{fwd,i}$).

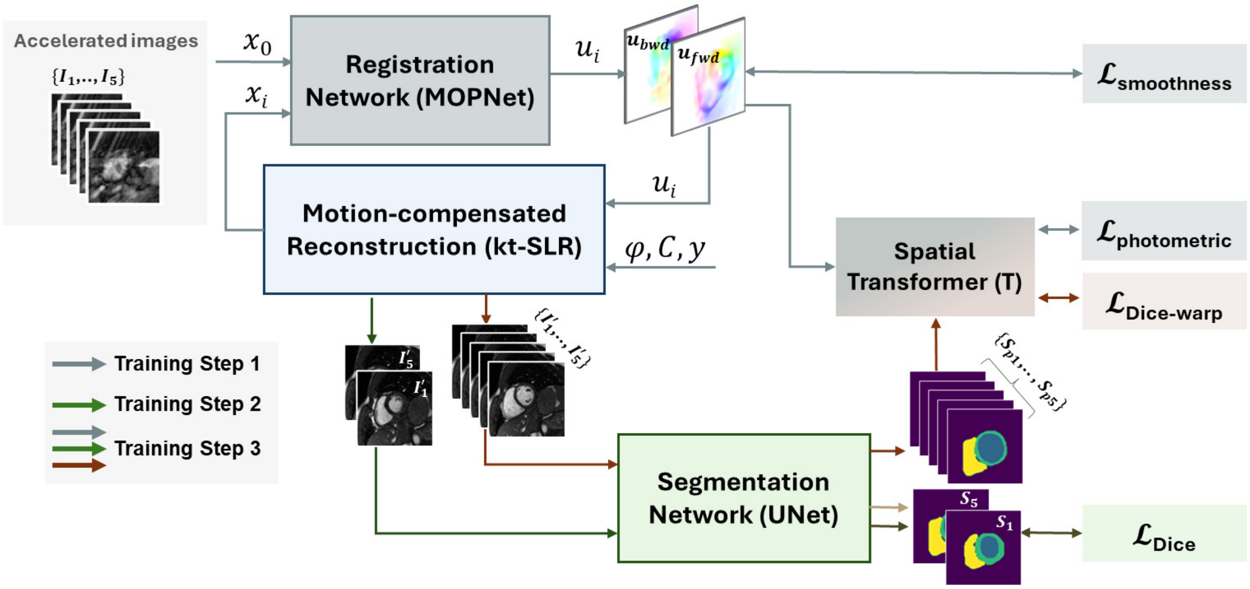

Figure S2: Overview of the joint learning of registration and segmentation from five successive $\{I_1, ..., I_5\}$ accelerated data. Initially, the registration network undergoes independent training utilizing a self-supervised strategy with the photometric $\mathcal{L}_{\text{photometric}}$ and smoothness $\mathcal{L}_{\text{smoothness}}$ losses on accelerated data. Subsequently, the segmentation network is trained with fully sampled data on manual available annotations S_1 and S_5 using a soft multi-class Dice loss $\mathcal{L}_{\text{Dice}}$. Both networks are then jointly optimized on fully sampled and accelerated data, integrating motion-compensated reconstruction to generate higher quality images $\{I'_1, ..., I'_5\}$ and mitigate undersampling artifacts. During this joint training phase, pseudo-labels $\{S_{p1}, ..., S_{p5}\}$ derived from the registration network are used to train the segmentation network via an additional multi-class Dice loss $\mathcal{L}_{\text{Dice-warp}}$, thereby leveraging information from unlabeled images.

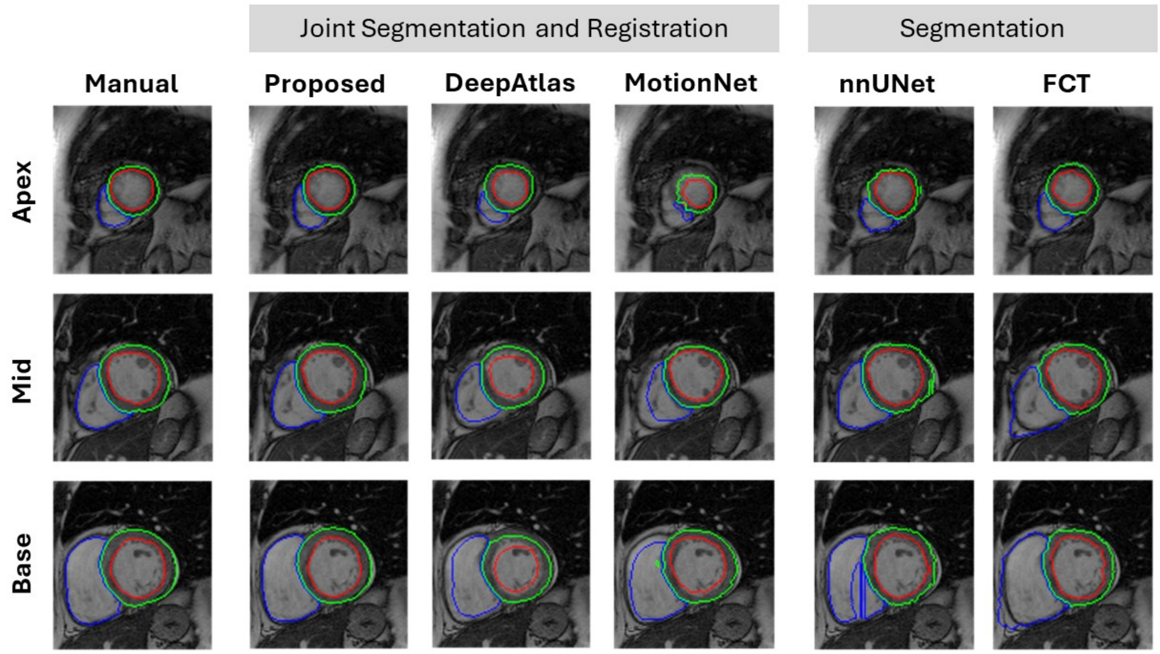

Figure S3: End-diastolic segmentation of the left and right ventricles, including apical, mid-ventricular, and basal segments overlaid on short-axis cine images. Results from the proposed method were compared to those of the segmentation networks (nnUNet[1], FCT[2]) and the joint segmentation and registration competing methods (MotionNet[3], DeepAtlas[4]).

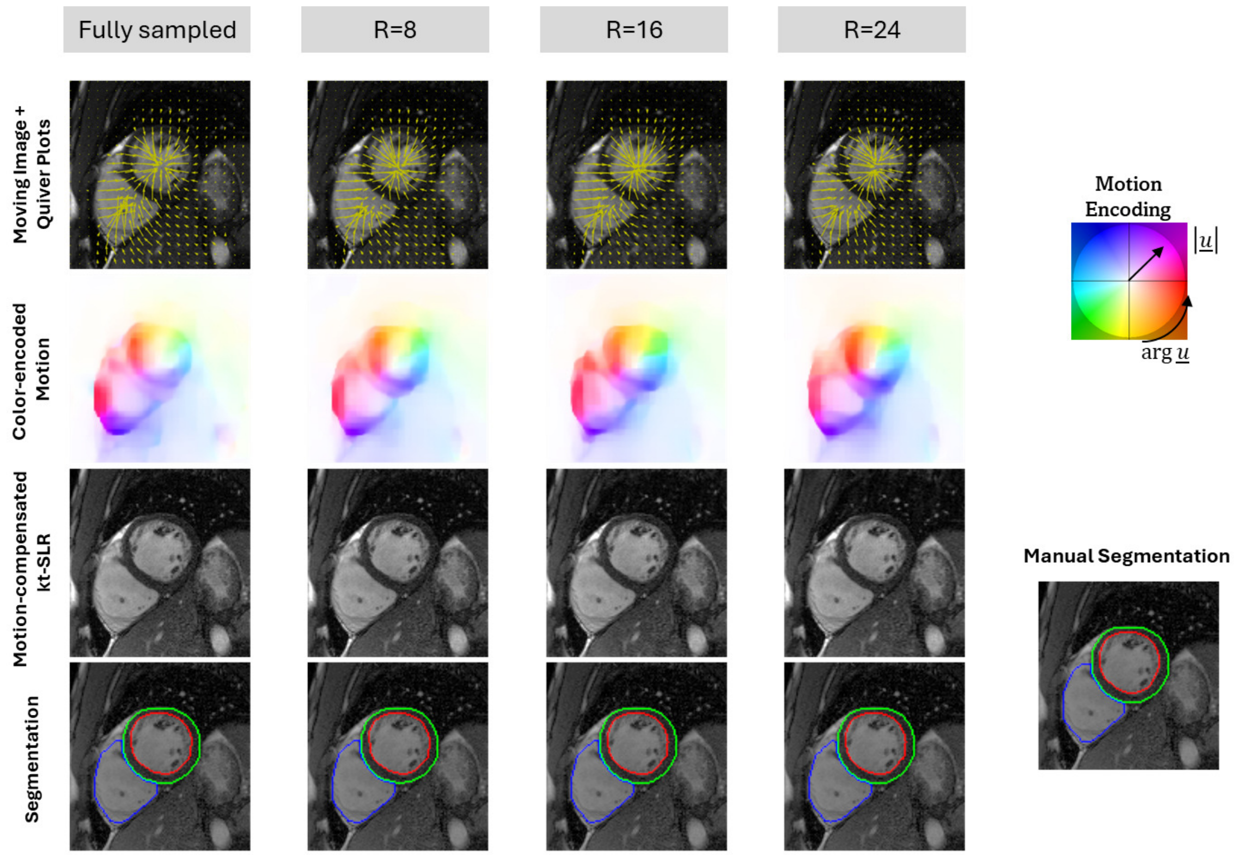

Figure S4: Representative motion estimates between end-diastolic and end-systolic cardiac cine frames, motion-compensated reconstruction, and segmentation at end-diastole obtained with the proposed framework from a healthy subject. Accelerated data is obtained with retrospective undersampling using the VISTA mask at three accelerations ($R = 8$, $R = 16$, and $R = 24$). Results are represented with quiver plots overlaid on the fully sampled moving image (first row), color-encoded [5] motion estimates (second row), motion-compensated reconstruction using kt-SLR (third row), and segmentation contours (last row) overlaid on the corresponding fully sampled images.

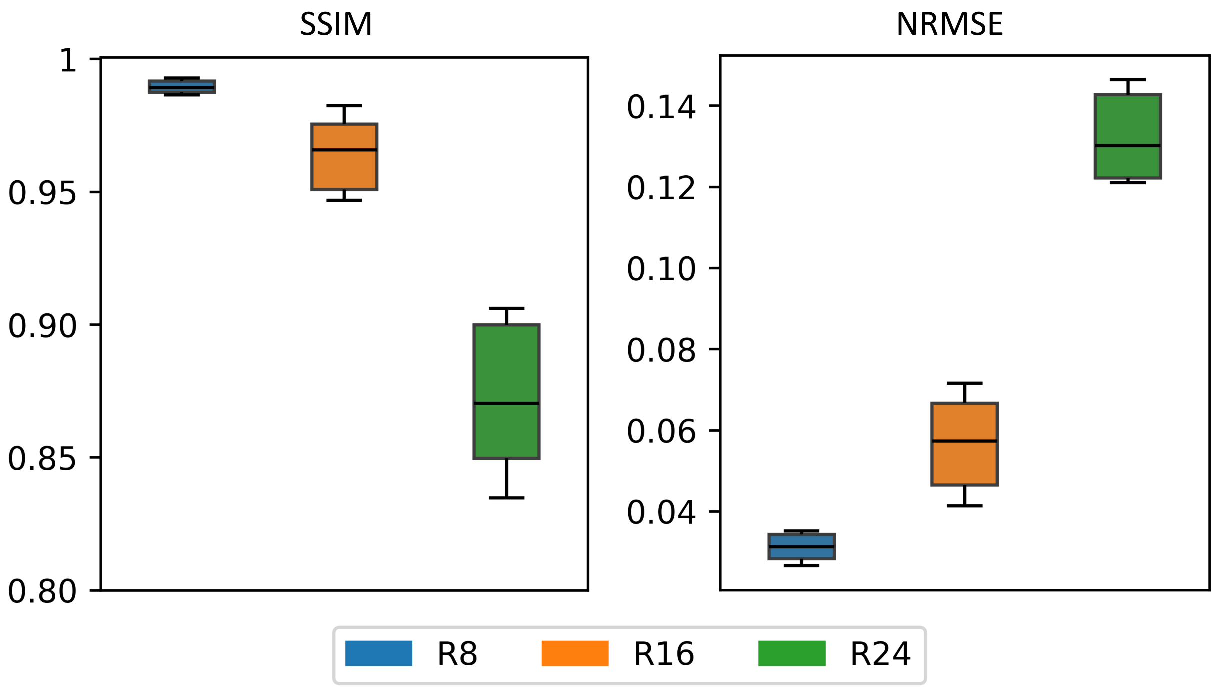

Figure S5: Quantitative analysis in terms of structural similarity index measure (SSIM) and normalized root mean squared error (NRMSE) between fully sampled data and proposed motion compensated kt-SLR reconstruction. Metrics are calculated for all subjects under $R = 8$, $R = 16$, and $R = 24$ retrospective undersampling with the VISTA mask. Results are depicted as box plots (horizontal line: median, box: 25% and 75% percentile) and calculated using fourfold cross-validation.

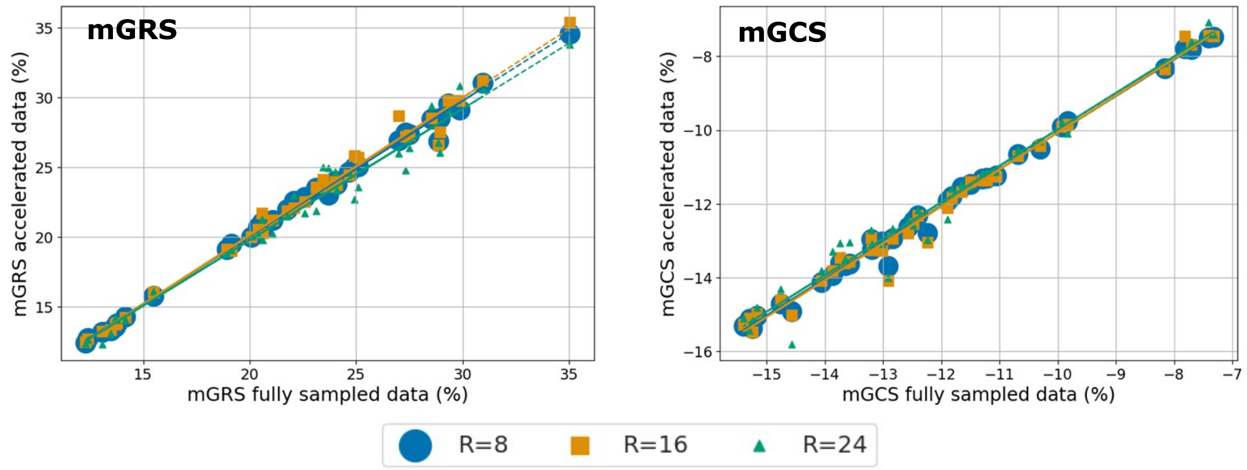

Figure S6: Linear regression plots illustrate performance differences between fully sampled and accelerated cases for mean Global Radial Strain (mGRS) and mean Global Circumferential Strain (mGCS). Our method maintains consistent strain measures across different acceleration factors.

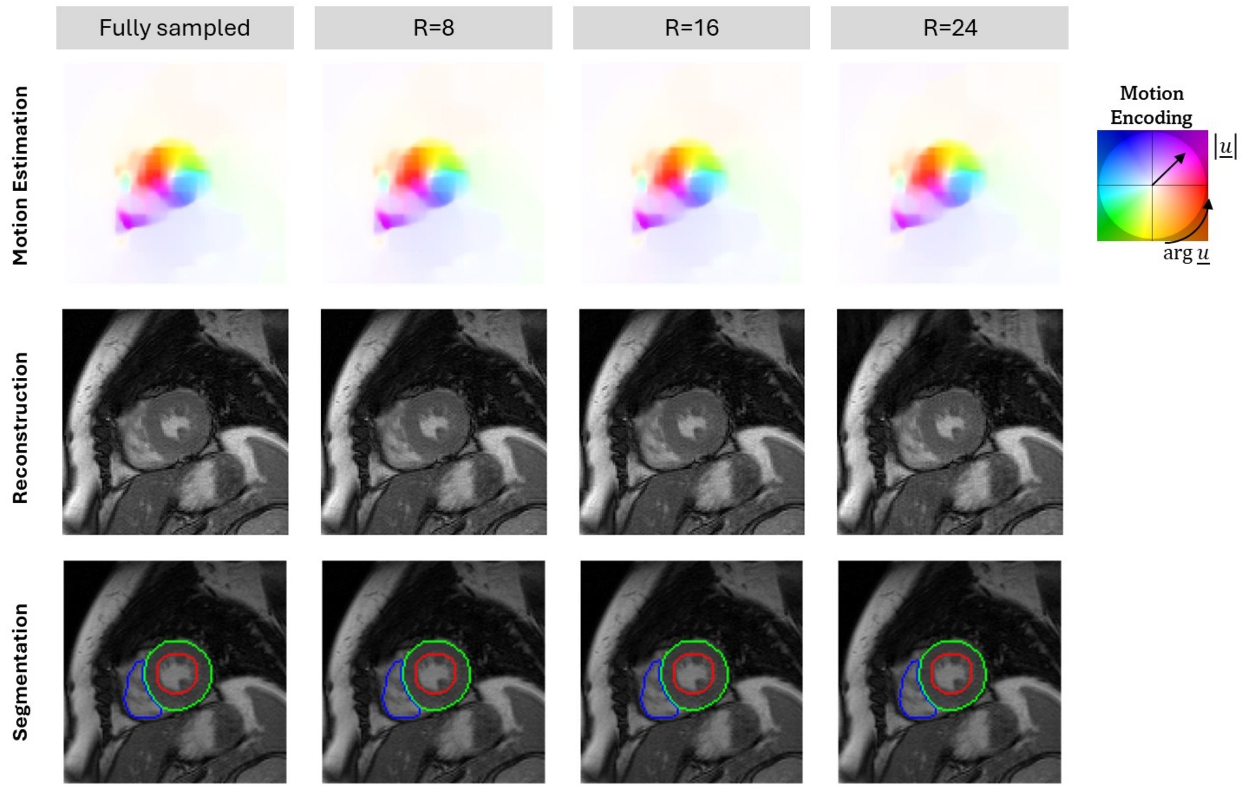

Figure S7: Representative motion estimates between end-diastolic and end-systolic cardiac cine frames, motion-compensated reconstruction, and segmentation at end-systole obtained with the proposed framework from post-contrast data of a patient with suspected cardiovascular disease. Accelerated data is obtained with retrospective undersampling using the VISTA mask at three accelerations ($R = 8$, $R = 16$, and $R = 24$). Results are represented as color-encoded [5] motion estimates (first row), motion-compensated reconstruction using kt-SLR (second row), and segmentation contours (last row) overlaid on the corresponding fully sampled images.

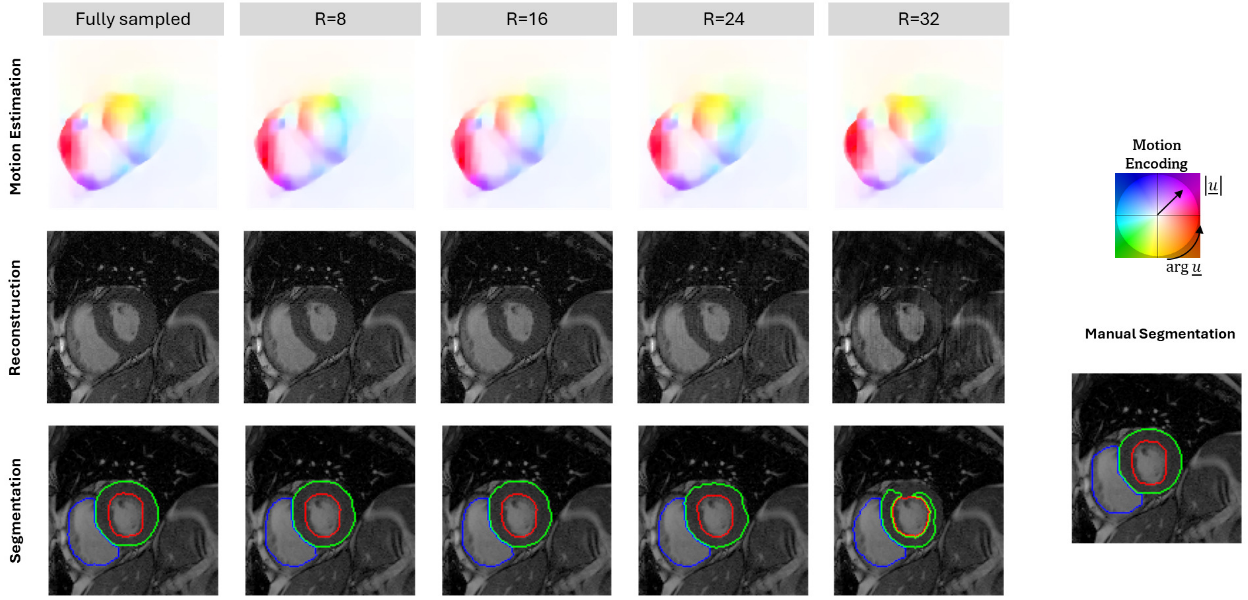

Figure S8: Representative results from a patient with suspected cardiovascular disease demonstrate motion estimation between end-diastolic and end-systolic frames of cardiac cine, motion-compensated reconstruction, and segmentation at end-diastole, which were all obtained using the proposed framework. Accelerated data was generated through retrospective radial sampling at acceleration factors of $R = 8$, $R = 16$, $R = 24$, and $R = 32$. Results are represented as color-encoded [5] motion estimates (first row), motion-compensated reconstruction using kt-SLR (second row), and segmentation contours (last row) overlaid on the corresponding fully sampled images.

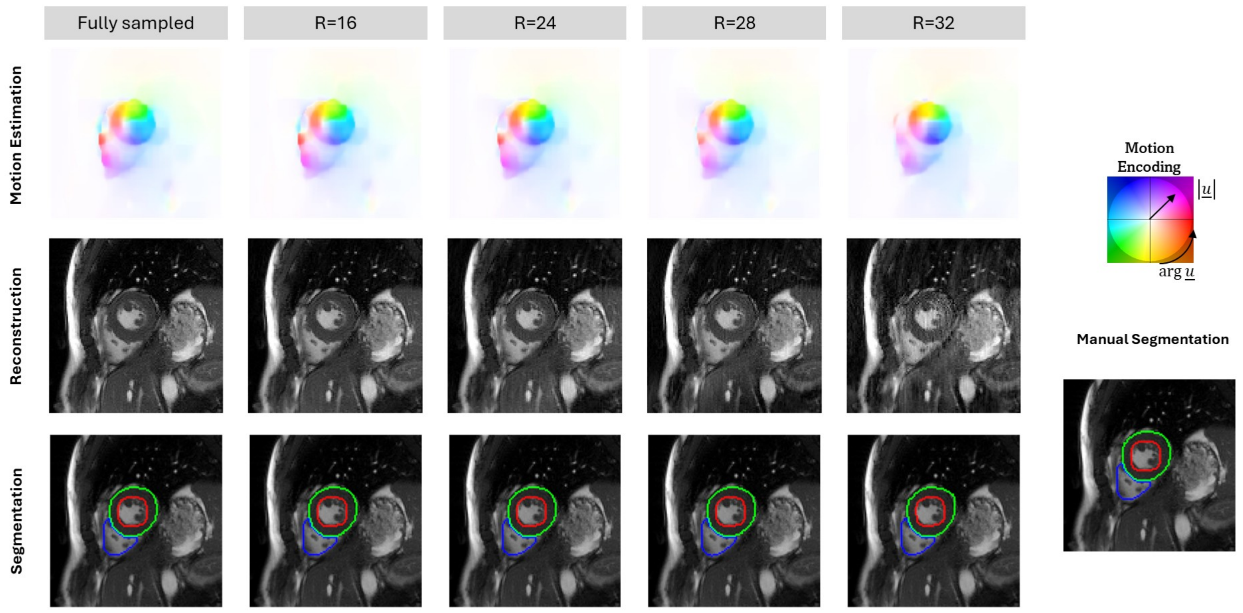

Figure S9: Representative motion estimates between end-diastolic and end-systolic cardiac cine frames, motion-compensated reconstruction, and segmentation at end-systole obtained with the proposed framework for a patient with complex Holt-Oram syndrome including congenital defects and pulmonary atresia. Accelerated data is obtained with retrospective undersampling using the VISTA mask at four accelerations ($R = 16$, $R = 24$, $R = 28$ and $R = 32$). Results are represented as color-encoded [5] motion estimates (first row), motion-compensated reconstruction using kt-SLR (second row), and segmentation contours (last row) overlaid on the corresponding fully sampled images.

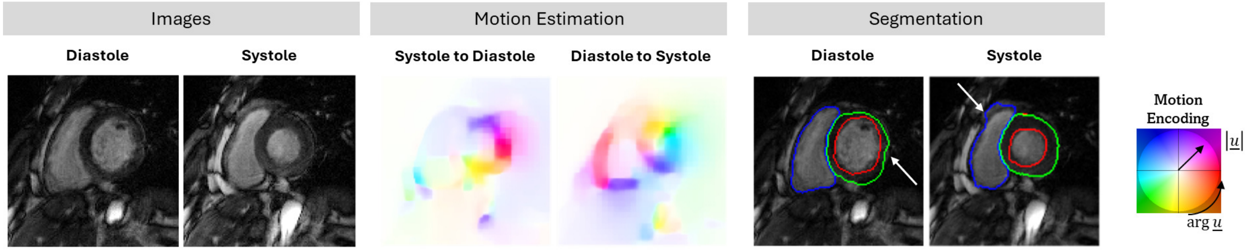

Figure S10: Image registration and segmentation results produced by our method on fully sampled example data from the CMRxRecon dataset, without any retraining or fine-tuning. The CM-RxRecon dataset features a higher spatial resolution than the training data. Color-coded motion estimations from systole to diastole and from diastole to systole are visualized [5]. Despite the domain shift in spatial resolution, MOPNet successfully captures the underlying cardiac motion patterns. While ground truth labels were unavailable, the segmentations showed minor discrepancies, suggesting potential improvement through fine-tuning. No major failures or outliers were observed, indicating robustness and promising generalizability.

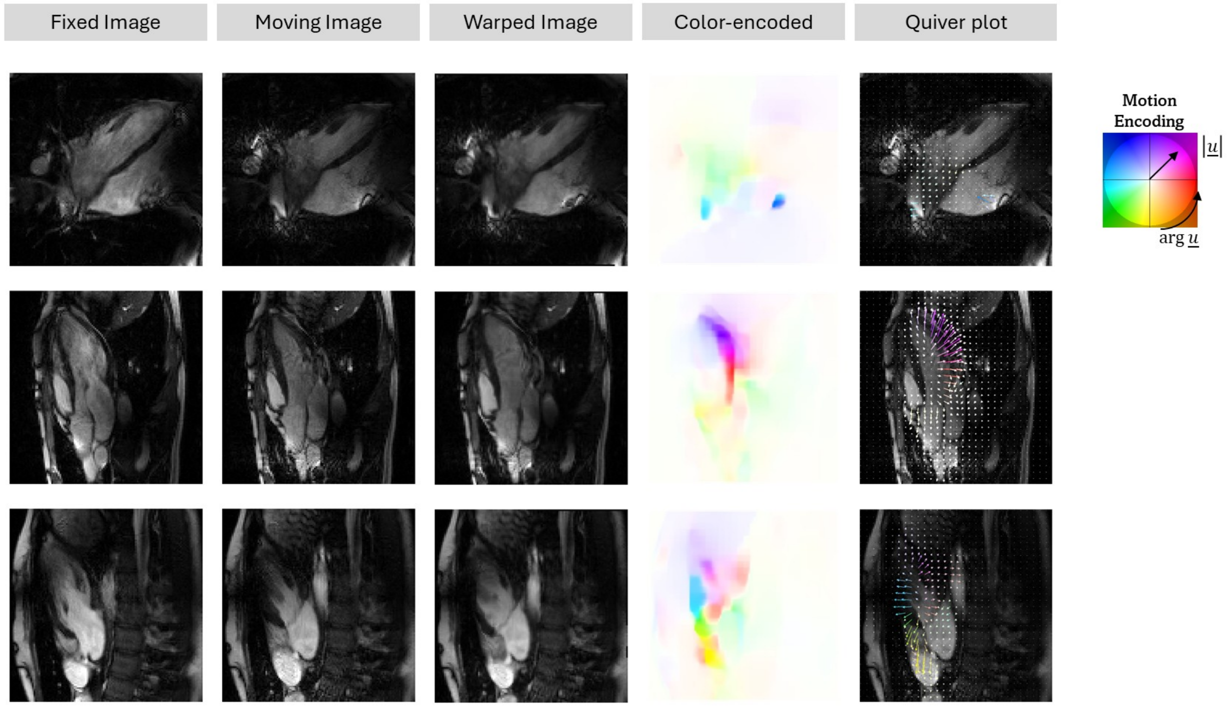

Figure S11: MOPNet-based motion estimation from systolic to diastolic frames on fully sampled long-axis 2-chamber, 3-chamber, and 4-chamber views from the CMRxRecon dataset. Motion fields are visualized as quiver plots, color-coded [5], and accompanied by error maps before and after registration. Although trained exclusively on short-axis data, MOPNet produces motion estimations consistent with the underlying relaxation patterns across previously unseen anatomical orientations.

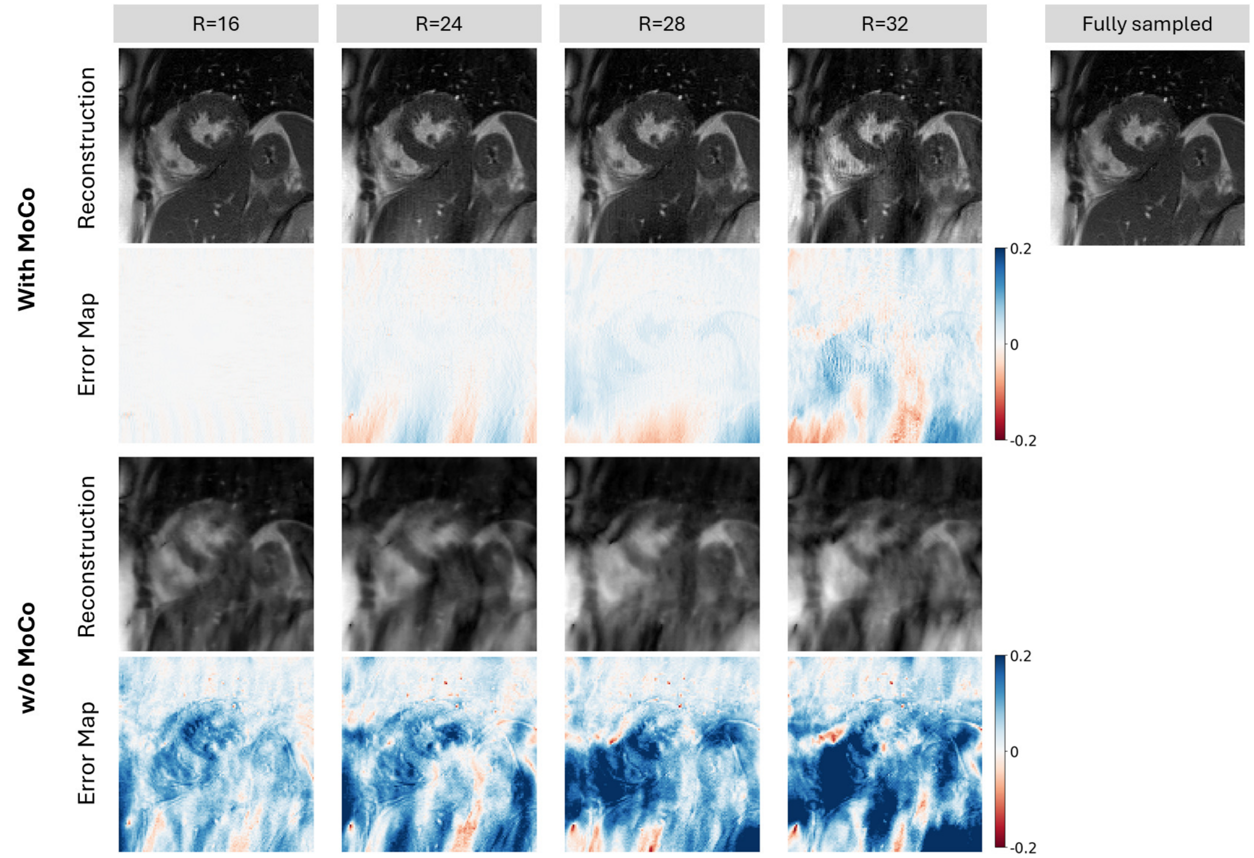

Figure S12: Reconstruction results for a patient with myocarditis using kt-SLR, shown alongside absolute error maps with respect to the fully sampled reference image. Reconstructions are presented both with and without motion compensation (MoCo) for acceleration factors of $R = 16$, $R = 24$, $R = 28$, and $R = 32$, using the VISTA sampling mask.

References

[1] Isensee Fabian, Jaeger Paul F, Kohl Simon AA, Petersen Jens, Maier-Hein Klaus H. nnU-Net: a self-configuring method for deep learning-based biomedical image segmentation. *Nature methods.* 2021;18(2):203–211.

[2] Tragakis Athanasios, Kaul Chaitanya, Murray-Smith Roderick, Husmeier Dirk. The fully convolutional transformer for medical image segmentation. In: :3660–3669; 2023.

[3] Qin Chen, Bai Wenjia, Schlemper Jo, et al. Joint learning of motion estimation and segmentation for cardiac MR image sequences. In: :472–480Springer; 2018.

[4] Xu Zhenlin, Niethammer Marc. DeepAtlas: Joint semi-supervised learning of image registration and segmentation. In: :420–429Springer; 2019.

[5] Baker Simon, Scharstein Daniel, Lewis James P, Roth Stefan, Black Michael J, Szeliski Richard. A database and evaluation methodology for optical flow. *International journal of computer vision.* 2011;92:1–31.
